# Searching for the Optimal Sampling Solution: Variation in Invertebrate Communities, Sample Condition and DNA Quality

**DOI:** 10.1371/journal.pone.0148247

**Published:** 2016-02-03

**Authors:** Martin M. Gossner, Jan-Frederic Struwe, Sarah Sturm, Simeon Max, Michelle McCutcheon, Wolfgang W. Weisser, Sharon E. Zytynska

**Affiliations:** 1 Technische Universität München, Terrestrial Ecology Research Group, Department of Ecology and Ecosystem Management, School of Life Sciences Weihenstephan, Hans-Carl-von-Carlowitz-Platz 2, 85354, Freising, Germany; 2 Zoological Research Museum Alexander Koenig, Adenauerallee 160–162, 53113, Bonn, Germany; Biodiversity Research Center, Academia Sinica, TAIWAN

## Abstract

There is a great demand for standardising biodiversity assessments in order to allow optimal comparison across research groups. For invertebrates, pitfall or flight-interception traps are commonly used, but sampling solution differs widely between studies, which could influence the communities collected and affect sample processing (morphological or genetic). We assessed arthropod communities with flight-interception traps using three commonly used sampling solutions across two forest types and two vertical strata. We first considered the effect of sampling solution and its interaction with forest type, vertical stratum, and position of sampling jar at the trap on sample condition and community composition. We found that samples collected in copper sulphate were more mouldy and fragmented relative to other solutions which might impair morphological identification, but condition depended on forest type, trap type and the position of the jar. Community composition, based on order-level identification, did not differ across sampling solutions and only varied with forest type and vertical stratum. Species richness and species-level community composition, however, differed greatly among sampling solutions. Renner solution was highly attractant for beetles and repellent for true bugs. Secondly, we tested whether sampling solution affects subsequent molecular analyses and found that DNA barcoding success was species-specific. Samples from copper sulphate produced the fewest successful DNA sequences for genetic identification, and since DNA yield or quality was not particularly reduced in these samples additional interactions between the solution and DNA must also be occurring. Our results show that the choice of sampling solution should be an important consideration in biodiversity studies. Due to the potential bias towards or against certain species by Ethanol-containing sampling solution we suggest ethylene glycol as a suitable sampling solution when genetic analysis tools are to be used and copper sulphate when focusing on morphological species identification and facing financial restrictions in biodiversity studies.

## Introduction

Most scientists in the field of biodiversity research and conservation agree that there is a great demand for the standardisation of biodiversity assessments [1–8]. Nevertheless the theoretical recommendations are yet not sufficiently implemented in monitoring campaigns across projects, institutes and countries. This is not least because standardisation has been optimised mostly within working groups rather than in between-group consultation, e.g. by workshops. For each individual work group, a change of method would mean reduced comparability to historic data.

Meta-analyses across studies have largely increased in importance in Ecology and provide great value in generalising consequences of various processes, e.g. land-use change and intensification [9,10], habitat loss and climate change [11]. The lack of standardisation, however, can also limit the comparability of studies across multiple research groups and projects across larger scales, and thus limit their ecological significance for such meta-analyses. The most promising way to achieve comparable sampling over long time periods, independent from the people involved and the specific weather conditions during sampling, is continuous passive sampling. This will also reduce the costs and time effort of long time monitoring, when compared to active collecting. Nevertheless, some potential biases have to be considered. For example, there is evidence that when placing a trap in the forest canopy, the position may have an effect [4], and it is clear that canopy traps will sample different species than traps placed on the ground. Less clear is how the use of various collection solutions within these methods might affect both the community composition and suitability of the samples for downstream processing (e.g. morphological or genetic analysis). Hence, there is an urgent need for comprehensive studies on the comparability of communities sampled using different sampling solutions.

For assessing arthropod diversity in forest ecosystems flight-interception traps are frequently used, in particular for beetles [9,12–16] and true bugs [17–22]. Beside trap design (see, e.g., [9]) sampling solution also differs between projects and this might be a big issue if groups or species are differently attracted by the various sampling solutions. Stoeckle et al. [5], for example, reviewed published studies with respect to different aspects of common sampling solutions, such as attractiveness, toxicity, evaporation, preservation of morphological characteristics and costs, which are all prevailing issues in biodiversity monitoring. They suggest differences in attractiveness between sampling solutions. The underlying data is, however, rather sparse and based on a few case studies using pitfall traps in single habitats and these are mostly published in local journals limiting visibility and generalisability. Nevertheless, similar effects might be expected for samples caught by flight-interception traps. Preservation of specimens for morphological species identification is another important issue. The quality of samples is expected to differ greatly between sampling solutions [5], but also on this topic only a few local pitfall trap studies exist.

Additionally, the effect of sampling solution might highly depend on the microclimatic conditions and the species present in a particular habitat. Thus, we expect to find different results when exposing traps in more stable microclimatic conditions at near ground level or in more fluctuating conditions, with higher extremes, in the forest canopy [23]. Moreover, forest types are known to provide different microclimatic conditions, i.e. broad-leaved forests differ greatly from spruce forests [24]. For a reliable estimation of the importance of sampling solution in biodiversity monitoring with flight-interception traps, field studies across those habitats and vertical strata are thus urgently needed.

DNA barcoding is increasingly used for species identification in biodiversity projects and barcode reference libraries are exponentially growing, e.g. for central European Coleoptera and Heteroptera [25,26]. Barcoding complements morphological based taxonomy and can improve rapid biodiversity assessments [27]. Moreover, the current and future value of specimens sampled by large biodiversity projects for molecular studies, e.g. on phylogenetics as well as population and conservation genetics, has increasingly been realised [28,29]. Successful DNA preservation methods such as rapid deep freezing, rapid drying or pure ethanol are often not practicable in big biodiversity projects due to the necessity for longer sampling intervals. Therefore it is important to also consider the consequences of other sampling solutions on DNA quality. Previous studies suggest that specimens sampled by different sampling solutions highly differ in their effects on DNA quality and genotyping reliability. These studies were, however, either conducted under laboratory conditions [5,30], used only one sampling solution [31] or used only a short exposition time to avoid evaporation issues when using ethanol [32] which is not practicable in most biodiversity projects.

In this study we tested the effects of sampling solution on the sample condition, DNA quantity and quality and different community parameters of sampled insects in a large continuous forest area, north of Freising, Germany. We therefore used a randomised block design with two different forest types (beech-dominated vs. spruce), three sampling solutions, two trap types (canopy and understory) and two collection jars per trap (top and bottom jar). In total 120 flight-interception traps were installed. We used three of the most commonly used sampling solutions in biodiversity monitoring studies: copper sulphate [17,33,34], ethylene glycol [35–37], and a mix of glycerine, ethanol and water (also known as Renner solution, excl. acetic acid; [38] [39,40]). According to Stoeckle et al. [5] ethylene glycol and Renner solution may actively attract species more than copper sulphate. Preservation of specimens is, however, expected to be better in ethylene glycol and Renner solution than in copper sulphate. Further, with regards to the cost of reagents, copper sulphate is very cheap, Renner moderate and ethylene glycol very expensive to use for large-scale studies.

In detail we asked the following questions:

1. Does condition of samples differ between the three sampling solutions and thus might affect further sample processing?
2. Do communities sampled by the three sampling solutions differ in terms of order abundance and diversity, species abundance and diversity and community composition?
3. Do effects of sampling solutions depend on forest type (beech vs. spruce), vertical stratum (canopy vs. understorey) or sampling jar (top vs. bottom)?
4. Does DNA quantity and quality of samples differ between the three sampling solutions and thus might affect subsequent genetic analyses?

## Materials and Methods

### Study site

This study was undertaken in the Wippenhauser Forest, North Freising, Germany (48.414°– 48.421°N / 11.714° - 11.732° E; Fig A in S1 File). The forest is part of the Upper Bavarian Tertiary Molasse-Hills. The climate is subatlantic to subcontinental with an annual average temperature of 7.4–7.7°C and a yearly precipitation of 800mm.

The studied forest sites are part of a continuous, managed forest of ca. 1500 ha which represents a historically old forest site. The natural forest communities are Luzulo-Fagetum and Galio odorati-Fagetum. Today, areas dominated by either Beech (*Fagus sylvatica*) or Spruce (*Picea abies*) trees exist within this forest (see Fig A in S1 File). The beech forests have an average tree age of 100 years (max. 165) and consists of 75% *Fagus sylvatica*, 23% *Quercus robur*, and 2% other broad-leaved tree species. They are managed as permanent forest leading to a two- to multi-layered forest. The spruce forests has an average age of 80 years and consists of 90% *Picea abies*, 5%, *Larix decidua* and 5% *Abies alba*, *Pseudotsuga menziesii*, and *Pinus sylvestris*. The forests are in a conversion stage aiming at increasing the proportion of broad-leaved trees.

### Experimental design

**Fig 1:**
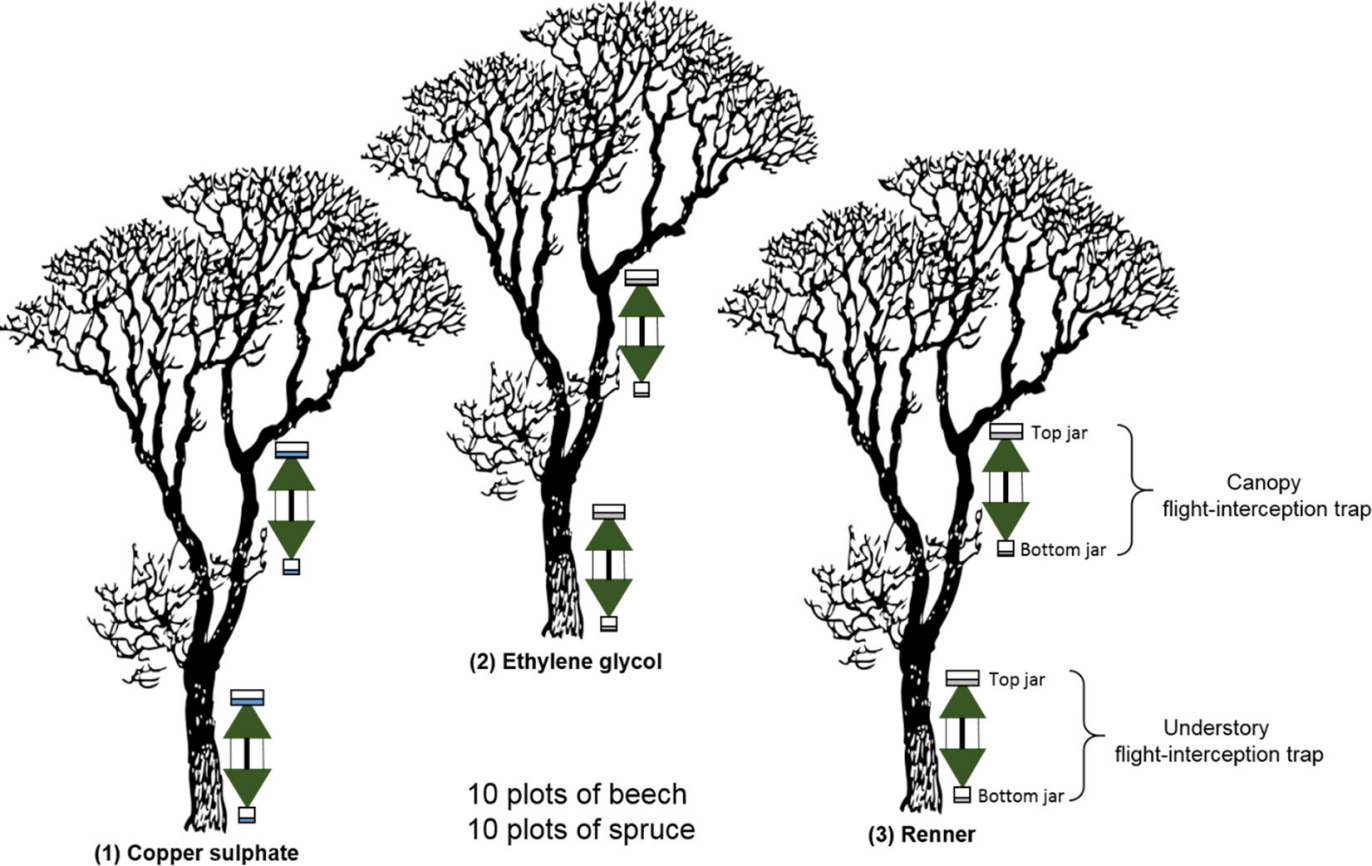
A diagram of the experimental design of one plot in the experiment. We used three trees, one for each sample solution, two traps per tree (canopy and understory) each with two jars (bottom and top). Distance between trees within each plot was five to ten meters. Ten plots were used per tree species (beech and spruce).

A randomised block design was used for this forest experiment, with two different tree species, three sampling solutions, two trap types (canopy and understory) and two collection jars per trap (top and bottom jar) (Fig 1). Ten repeats were made, leading to a total of 60 trees (30 of each species) with 120 traps and 240 collection jars. Each experimental block consisted of one forest plot with three trees, one each for the three sampling solutions. Thus, each forest plot contained one repeat per sampling solution.

### Experimental set-up

The forest plots were at least 50 m from each another with a maximum distance of 1400 m between plots. Ground dead-wood volume (mean ± SE; beech: 29.66±6.22 m^3^h^-1^, spruce: 42.42±6.83 m^3^h^-1^) and Shannon plant diversity per plot was lower in beech than in spruce forests (beech: 1.20±0.09, spruce: 1.38±0.06). Within each plot, three similarly sized trees (beech dbh: 66 ± 2 cm, height: 34.9 ± 0.8 m; spruce dbh: 48 ± 1 cm, height: 31.8 ± 0.5 m) were chosen that were located at least 5 m, but no more than 10 m, from each other. Crown volume was higher in beech (511±144 m^3^) than in spruce trees (199±19 m^3^), but crown dead-wood volume did not differ between tree species (beech: 4.38.±2.48 m^3^, spruce: 4.44±1.05 m^3^).

Flight-interception traps (FITs) were used to collect invertebrates. They consisted of crossed pairs of 40 cm x 60 cm sized transparent plastic shields with funnels and sampling jars attached at the top and the bottom (see [13] for general trap design). Three different sampling solutions (1) copper sulphate (CuSO_4_ 3%), (2) ethylene glycol (50%) and (3) ethanol (40%) / glycerine (25%) / water (35%) (also known as “Renner solution” and hereafter referred to as such, but excl. acetic acid) were used; chosen due to the current and potential use in long-term, large-scale biodiversity experiments. Copper sulphate is, for example, used in the long-term German Biodiversity Exploratories project [33,41], Renner in the long-term studies of forest nature reserves in Hessen, Germany [39,40], and ethylene glycol in the European Beech Forest for the Future project (BeFoFu; http://www.befofu.org/; [37]) within BiodivERsA network of the EU 7th Framework Programme for Research. One tree in each plot was used per sampling solution (i.e. total of 3 trees per plot) with two FITs per tree (canopy and understory). The canopy traps were installed using a crossbow to shoot a string over a suitable branch in the centre of each tree crown (height: beech: 15.1±1.0; spruce: 18.5±0.7). The traps were secured with rope. The understory trap was installed next to the tree trunk, by suspending from rope ensuring the bottom jar of the trap was not touching the forest floor (height 1.5 m). The traps were installed in early May and emptied twice, once in late-May (three weeks after installation) and again at the end of June (seven weeks after installation). The sampling solution was replaced after the first collection in all jars and all samples were immediately transferred to 70% ethanol in the field. The pH of the solution of all samples collected in June, and a subset from May, was also measured. The pH was measured three times using a pH meter (WTW pH 320, Germany) and the average value calculated. A dilution series test was made to show how the pH of the sampling solutions can change with increased water (e.g. from rain). Each sampling solution was tested at 100%, 50%, 25%, 20%, 5% and 1% of its original strength, diluted with double-distilled water.

### Invertebrate identification and classification

All samples were sorted to arthropod order level in the laboratory. Subsequently, all Coleoptera and Hemiptera: Heteroptera were identified to species level either by one of us (Heteroptera: MMG) or by a taxonomic specialist (Coleoptera). FIT's are widely used for sampling these taxonomic groups because they give a representative sample of their communities [42].

Beetles were classified based on their feeding ecology and habitat requirements. We classified all sampled species into feeding guilds (herbivores excl. xylophages, carnivores, mycetophages-fungi, mycetophages-mould, decomposers-wood, decomposers excl. wood), habitat guilds (ground dweller, eurytopic, vegetation, rotten substrate/nests/fungi-excl. wood) [43] and more specific dead wood substrate guilds (according to [44]): old dead wood (od-dweller), fresh dead wood (fd-dweller), wood mould and specific dead wood structures (rh- and s-dweller), wood fungi (fu-dweller). Among saproxylics the feeding guilds mycetophages, xylophages and carnivores were distinguished. We also measured body sizes of species as functionally meaningful trait [45].

### Measure of quality for morphological species determination

During sorting, all samples from the June collection were classified according to the conditions of the insects with respect to mould and completeness of the insects. This was used as a measure of quality for morphological species determination. Values ranged from 0.75 (excellent condition, no mould and insects all complete) to 3.25 (totally mouldy and insects largely fragmented) in steps of 0.25. Details are given in the S2 File.

### Species identification through DNA barcoding

#### Preliminary study (older samples from 2008–2011)

A preliminary study was undertaken to determine an optimal DNA extraction method (Salting Out [46] or Phenol-Chloroform [47]) on previously collected samples from four sampling solutions (copper sulphate, glycerine, ethanol and ethylene glycol). Five samples from seven Coleoptera species (collected 2009–2011) were used per sampling solution and extraction method (S3 File). Recovered DNA yield was consistently higher using the Salting-Out method [46], than the Phenol-Chloroform [47], and this was chosen for future sample extraction.

#### Current study samples

Ten species were chosen from the collected individuals to perform barcoding sequencing on, across the different sampling solutions. They were *Agathidium seminulum* (Linnaeus 1758) (Coleoptera, Leiodidae); *Athous subfuscus* (O. F. Muller 1764) (Coleoptera, Elateridae); *Corticaria abietorum* (Herbst 1783) (Coleoptera, Latridiidae); *Corticarina lambiana* Sharp 1910 (Coleoptera, Latridiidae); *Cychramus variegatus* (Herbst 1792) (Coleoptera, Nitidulidae); *Metacantharis discoidea* (Ahrens 1812) (Coleoptera, Cantharidae); *Plectophloeus fischeri* (Aube 1833) (Coleoptera, Staphylinidae); *Rhynchaenus fagi* (Linnaeus 1758) (Coleoptera, Curculionidae); *Trixagus dermestoides* (Linnaeus 1766) (Coleoptera, Throscidae); and *Psallus varians* (Herrich-Schäffer 1841) (Hemiptera, Miridae). Fifteen individuals from each species were sampled, five from each of the three sampling solutions. All specimens of one species were taken from one forest type and one vertical stratum to minimize microclimatic bias. Because there were not enough specimens of any species across stratum and forest type effects of microclimatic differences on DNA quantity and quality could not be tested.

Each individual was first washed with 70% ethanol and the ethanol allowed to evaporate. For the majority of species the whole insect was used for the DNA extraction, except for *Athous subfuscus*, *Cychramus variegatus* and *Metacantharis discoidea* for which individuals were larger and thus leg material was used. The weight of material per sample used was recorded to allow recovered DNA yield to be calculated. DNA was extracted using the Salting-Out method [46]: insect tissue was homogenised in 300 μl TNES buffer (50 mM Tris-HCl pH8, 20 mM EDTA, 400 mM NaCl, 0.5% SDS) with 5 μl of Proteinase K (20 mg/μl) and incubated overnight in a water bath at 37°C. Then 85 μl of 5 M NaCl was added, vortexed and the sample centrifuged at 13,000 rpm for 10 min. The supernatant was placed into a new tube and 400 μl 100% Ethanol added and the sample kept at -20°C for a minimum of one hour for DNA precipitation. After centrifugation at 13,000 rpm for 20 min the DNA pellet was twice washed in 70% ethanol and air-dried (to evaporate remaining ethanol) before being resuspended in TE buffer (10mM Tris-HCl (pH 8.0), 1 mM EDTA), and stored at -20°C. DNA concentration (ng/μl) was measured using a Quantus™ Fluorometer (Promega), and the yield (ng/mg) calculated.

DNA integrity was determined using a Fragment Analyzer (Advanced Analytical Technologies, Inc., Ames, IA) with its current software version 1.0.2. Samples were prepared following the manufacturers specifications for the use of the high sensitivity kit (DNF-488 High Sensitivity Genomic DNA Analysis Kit). Whenever needed the DNA concentration was diluted to a maximum of 5 ng/μl to level sample concentrations. Smear analyses were conducted using the provided software PROSize 2.0 Software Version 1.3 to quantify the proportion of higher genomic DNA (1000 bp– 20000 bp) to the rest of the sample and to identify the average size of the measured sequence lengths. The 1000 bp cut-off was chosen to include intact DNA as well as partially degraded DNA [48] into the measurement. Partially degraded DNA still maintains potential to lead to successful identifications due to the limited length of CO1 and the abundant occurrence of mtDNA in the cells. Furthermore 1000 bp is also the threshold to which unwanted RNA might be present in the extraction (User Guide DNF-488 High Sensivity Genomic DNA Analysis Kit 2014MAR11). In this approach it can be considered as useless as heavily degraded DNA.

A 658 bp fragment of the CO1 gene was amplified using universal primers LCO1490 (5’GGTCAACAAATCATAAAGATATTGG 3’; Folmer et al. [49]) and C1-N-2191 (5’CCCGGTAAAATTAAAATATAAACTTC 3’; Simon et al. [50]). PCR were performed in 20 μl volume, with 1.5 μl template DNA, 1 U Bioline MyTaq DNA polymerase, 4 μl 5X Bioline MyTaq Reaction Buffer, 0.5 μM primer F, 0.5 μM primer R. Following optimisation in the preliminary study using a gradient PCR, a touchdown PCR (53–48°C) was used on a thermocycler (BIOER Lifetouch ™): 95°C 5 min, followed by 10 cycles of 94°C 15 s, 53–48°C 30 s (-0.5°C per cycle), 72°C 30 s, then 25 cycles of 94°C 15 s, 48°C 30 s, 72°C 30 s, finally ending with 72°C for 6 min and then cooled to 4°C. The results were visualised on a 1.2% agarose gel using DNA Stain G (SERVA, Germany) and visualised using a gel documentation system (Intas Gel-Stick, Royal Biotech, Germany). An 8 μl aliquot of the PCR product was then cleaned using ExoSap (0.1 U FastAP, 0.4 U Exonuclease I), and incubated at 37°C for 30 min followed by 80°C for 15min in a thermocycler. Cleaned products were sequenced by Macrogen. Samples that failed to produce a PCR band or sequencing product were repeated.

### Sequence processing

The resulting sequence reads were fully processed in Geneious version 7.1.9, [51]. Raw reads were assembled (de novo assemble) using the geneious assembler on highest sensitivity default mode (Allow Gaps = true; Word length = 10; Index word length = 10; Maximum mismatches per reads = 50%; Maximum ambiguity = 16; Maximum gap size = 5). Read directions (Forward and Reverse) were checked and corrected if necessary. Primer sections were trimmed. From each of the assembled reads consensus sequences were calculated, setting the threshold for matching bases at 100% identical, allowing ambiguities according to the IUPAC Ambiguity Code to encode for ambiguous positions. The resulting sequences were assigned into the categories High, Medium and Low for an overall sequence quality rating. Each category is a result of the evaluation of further quality thresholds. A sequence of the category High is allowed no ambiguities and a minimum length of 300 bases. 90% of the bases need to have a phred score of ≥40. Only 10% of the bases are allowed to have a phred score below 20. For Medium classification a maximum of 5 ambiguities is accepted. The minimum sequence length is again 300 bases. 80% of the bases need to have a phred score ≥40 and 15% are allowed to have a phred score below 20. Any sequence that did not fit these criteria was assigned Low.

Sequences were then cross-checked for matches in BOLD and NCBI databases. In BOLD the offered BOLD Identification System (IDS) for animal identification was used [52]. In NCBI the query sequences were analysed using BLASTN 2.2.31+ [53]. The data (available matches and their corresponding values: similarity (BOLD) and Max score and Identity (NCBI)) were then transferred into a table.

### Data analysis

All analyses were done in R (version 3.2.0) [54] using R studio (version 0.98.977). The data were analysed using eight response variables: (1) sample condition, (2) order richness, (3) order diversity, (4) order abundance, (5) Coleoptera species richness, (6) Coleoptera species diversity, (7) Hemiptera: Heteroptera species richness, and (8) Hemiptera: Heteroptera species diversity. For the order abundance data, the nine orders with greater than 500 individuals collected across all treatments (99.0% of the data) were analysed. The diversity was calculated as the exponential Shannon diversity using the vegan package [55]. Linear mixed effects models, using R package nlme [56] were used to determine the effect of the fixed factors: tree species, sampling solution, trap type (canopy or understory) and collection jar (top or bottom), and the random effects used were plot, sampling solution, trap type and jar to account for the hierarchical structure of the data set. For the order abundance model, an additional fixed effect of ‘order’ was used in the model. Full models were fit first, including all interactions (i.e. lme(<Response variable> ~TreeSpecies*Solution*TrapType*Jar, random = ~1|Plot/Solution/TrapType/Jar)), and then each model was simplified by removing the most non-significant term first using a backwards selection procedure. Post-hoc contrasts from the models in R are presented to show differences among levels within a factor. The pH of copper sulphate varied between the top and bottom jars, thus a further model to determine if the effects on sample condition were dependent on the changing pH was run on the copper sulphate data. Reduced (minimum adequate models) are presented in the results.

For a more detailed analysis of changes in community composition we used RLQ analysis [57] for an ordination of species, species traits and sites on the main environmental gradients (package ade4 in R; [58]). We used a fourth-corner analysis [59,60] as statistical test of the relation of the biological traits and the environmental variables through the link of a community data table. In the RLQ analyses the relationships between species traits (Q) and environmental variability (R) are revealed by maximizing the congruency between three data tables: Beetle abundance data (L-matrix), traits data (Q-matrix), and environmental data (R-matrix).

The genetic barcoding data was analysed using general linear models. The number of successful identifications was analysed using a generalized linear model with quasibinomial error distribution. The response variables was identification success (0,1) and the explanatory variables were species and sampling solution (and the interaction). To analyse DNA yield and DNA quality (average fragment length (bp) and concentration of DNA above 1000bp (ng/μl)) variation as response variables we used linear models with normal error distributions. The DNA yield data was log-transformed to achieve normal errors, and the explanatory variables were species and sampling solution (and the interaction) for each response variable. A second model, for each response variable, was run using the available pH and sample condition data (N = 109, from 150 total). The variables of DNA yield and quality were correlated and so individual models using generalised linear mixed effects models (binomial error) with species and solution as a random factor were used to assess the influence of these variables on the barcoding success. Full models were fit first, including all interactions and then each model was simplified by removing the most non-significant term first using a backwards selection procedure.

## Results

The traps collected 76,588 individuals from 29 orders (Table 1); additionally we sampled 5938 holometabolic larvae which were excluded from further analyses. Nine orders contained more than 500 individuals (Acari, Araneae, Coleoptera, Collembola, Diptera, Hemiptera, Hymenoptera, Psocoptera, Thysanoptera; Table A in S4 File).

**Table 1:** Summary of the results for order richness, diversity and abundance.

	Order richness			Order diversity			Order abundance		
Source	df	F	P	df	F	P	df	F	P
Order		na			na		8,1888	12.08	**<0.001**
Tree species	1,18	0.22	0.647	1,18	3.01	0.100	1,18	0.99	0.333
Sampling solution	2,38	0.93	0.404	2,38	2.38	0.107	2,38	0.94	0.401
Trap type	1,58	16.86	**<0.001**	1,58	18.00	**<0.001**	1,59	0.20	0.658
Jar	1,119	88.43	**<0.001**	1,118	33.55	**<0.001**	1,119	18.91	**<0.001**
Tree species x Trap type	1,58	4.50	**0.038**	1,58	4.20	**0.045**	-	-	-
TrapType x Jar	1,118	-	-	1,118	10.18	**0.002**	-	-	-
Order x Tree Species		na			na		8,1888	2.92	**0.003**
Order x Trap type		na			na		8,1888	3.47	**<0.001**
Order x Jar		na			na		8,1888	4.15	**<0.001**

Notes:—means the term was not retained in the minimum adequate model. All interactions (including the 4-way between all main effects) were tested, ones not shown were not retained in the minimum adequate model. Significant (P<0.05) terms are highlighted in bold. Order abundance tests the nine orders with at least 500 individuals (99.0% of total). ‘na’ means the term was not included in the model.

### Condition of the samples

**Fig 2:**
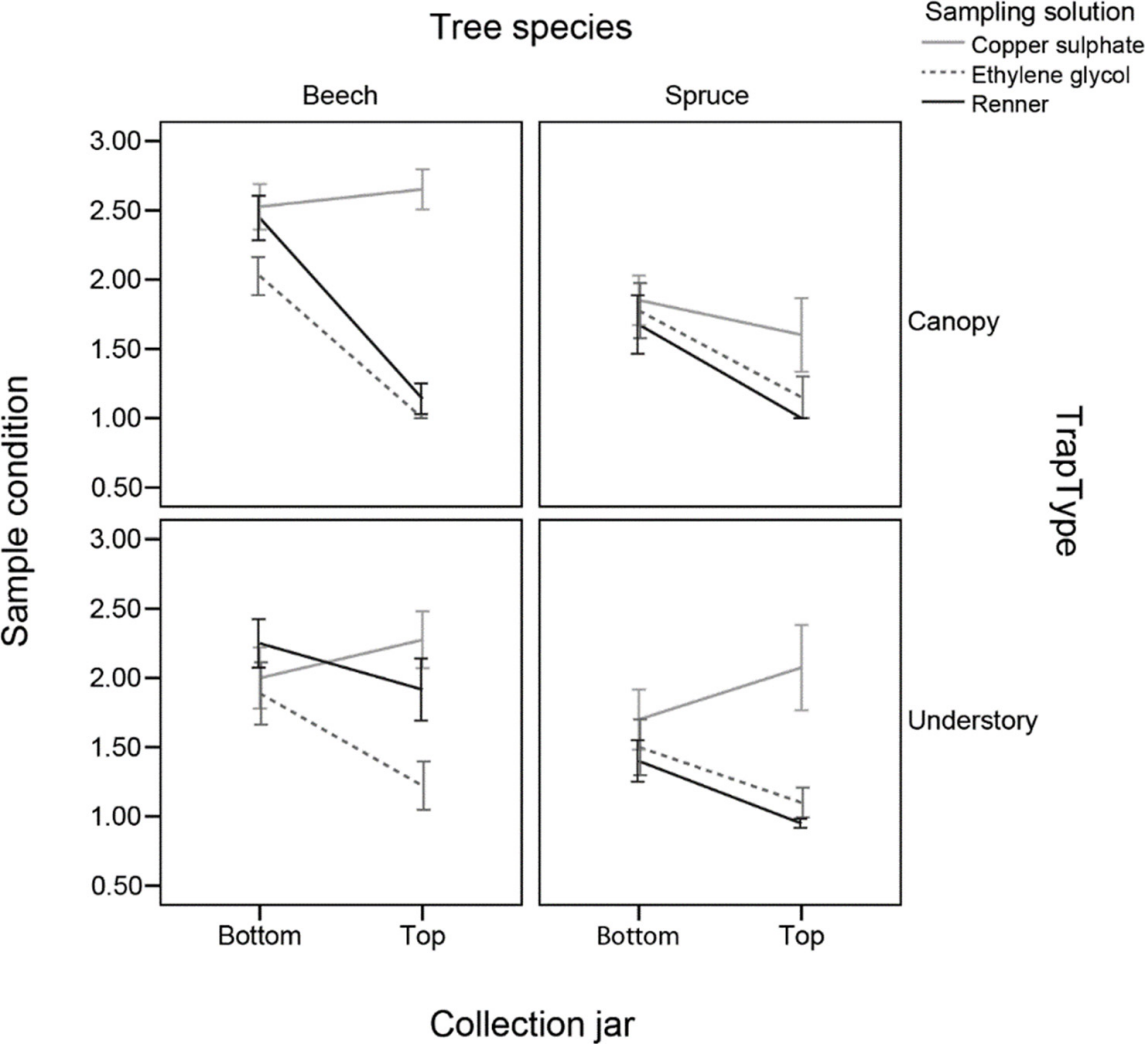
The condition of the samples across the different treatments. Higher values indicate worse condition. Error bars represent ±1SE.

The condition of the samples was found to be influenced by a number of treatment factors, including a 3-way interaction between tree species, sampling solution and trap type (F_2,54_ = 4.69, P = 0.013). Here, the copper sulphate sampling solution samples were observed to be in a worse condition than samples from the other solutions, and this is most apparent in the top jars of traps–particularly in canopy traps on beech and understory traps on spruce (Fig 2, Table A in S5 File). The pH of the copper sulphate samples was lower in the top than the bottom jars of the traps (F_1,39_ = 926.4, P<0.001), likely due to dilution of the bottom jars from rain (Fig A in S5 File); the pH of the other solutions was constant across the jars. However, neither the pH (F_1,38_ = 1.75, P = 0.193) nor the jar (F_1,38_ = 1.24, P = 0.272) affected the condition of the samples and as such the effect of sampling solution on sample condition was not linked to the pH.

### Order level

We found no effect of sampling solution on richness, diversity or community composition at the order level (richness: F_2,38_ = 0.93, P = 0.404; diversity: F_2,38_ = 2.38, P = 0.107; composition: R = -0.001, P>0.9). However, there was a higher order richness and diversity in the understory traps than the canopy traps (Table 1). Order composition also differed between trap type (R = 0.110, P<0.001), as understory traps collected more Acari, Isopoda and Orthoptera, whereas the canopy traps collected more Diptera, Mecoptera and Megaloptera. The effect of trap type also differed dependent on the tree species (Tree species x trap type interaction; Table 1); there was a greater difference in order richness and diversity between the understory and canopy traps for spruce than for beech. In general, the bottom jar collected more individuals than the top (Table 1) but a significant interaction between trap type and jar (Table 1) showed that the difference was larger in the understory than the canopy traps.

We analysed the abundance of nine orders, in which more than 500 individuals were collected in total, with respect to the treatments (Table 1). We found that the sampling solution did not alter the abundance of these orders, and all other fixed effects were significant as interaction effects (Table 1). The abundance of the different orders changed dependent on: the tree species, with spruce having more Diptera (post-hoc test: t = 2.49, P = 0.017) and beech more Thysanoptera (t = 2.36, P = 0.018); the trap type, with canopy traps collecting more Diptera (t = 4.74, P<0.001); and, the jar, with the bottom jar collecting more individuals overall, but specifically more Diptera (t = 2.49, P = 0.017).

### Species level analyses

#### Species richness and diversity

We collected 5,432 individuals of Coleoptera (Table A in S4 File), of which 5,278 could be identified to 326 species. The most abundant species were *Xyleborus germanus* (651 individuals), *Athous subfuscus* (611 individuals), *Eusphalerum sorbi* (505 individuals) and *Rhynchaenus fagi* (368 individuals). The samples from spruce were more species rich, with 243 species identified compared to 182 from beech. The species richness of the samples differed among the treatment factors with a three-way interaction between tree species, sampling solution and jar (Table 2); this was primarily driven by an increase in species richness in the bottom jars of understory traps containing the Renner sampling solution, to a greater extent in spruce than beech (Fig A in S6 File). However, this 3-way interaction term was not significant for the species diversity, where sampling solution was not present in any significant terms within the final model (Table 2).

**Table 2:** Summary of species richness and Shannon diversity results for Coleoptera species.

		Species richness			Species diversity	
Source	df	F	P	df	F	P
Tree species	1,18	12.29	**0.003**	1,18	17.59	**<0.001**
Sampling solution	2,36	1.31	0.284	2,38	1.23	0.303
Trap type	1,56	15.65	**<0.001**	1,58	6.22	**0.016**
Jar	1,86	753.67	**<0.001**	1,91	460.56	**<0.001**
Tree species x solution	2,36	0.71	0.498	-	-	-
Tree species x trap type	1,56	9.14	**0.004**	1,58	8.61	**0.005**
Solution x trap type	2,56	0.43	0.650	-	-	-
Tree species x jar	1,86	6.83	**0.011**	1,91	8.19	**0.005**
Solution x jar	2,86	3.07	0.051	-	-	-
Trap type x jar	1,86	0.47	0.495	-	-	-
Tree species x solution x jar	2,86	3.11	**0.050**	-	-	-

Notes:—means the term was not retained in the minimum adequate model. All interactions (including the 4-way between all main effects) were tested, ones not shown were not retained in the minimum adequate model. Significant (P<0.05) terms are highlighted in bold.

We collected 3,468 individuals from the order Hemiptera, 164 from the sub-order Heteroptera were identified into 32 species. Due to the lack of Heteroptera in the top jars, the data was combined from the bottom and top jars of each trap. The sampling solution influenced Heteroptera species richness (F_2,27_ = 3.33, P = 0.051) and diversity (F_2,27_ = 3.66, P = 0.039), with the Renner solution yielding fewer species and a reduced diversity than the other two solutions. The species richness and diversity of the samples did not differ among the tree species (species richness F_1,17_ = 0.019, P = 0.892; diversity F_1,17_ = 0.09, P = 0.771), or trap type (species richness: F_1,25_ = 0.56, P = 0.462; species diversity F_1,25_ = 1.21, P = 0.281). We found no interactions between the treatment factors.

#### Community composition

**Fig 3:**
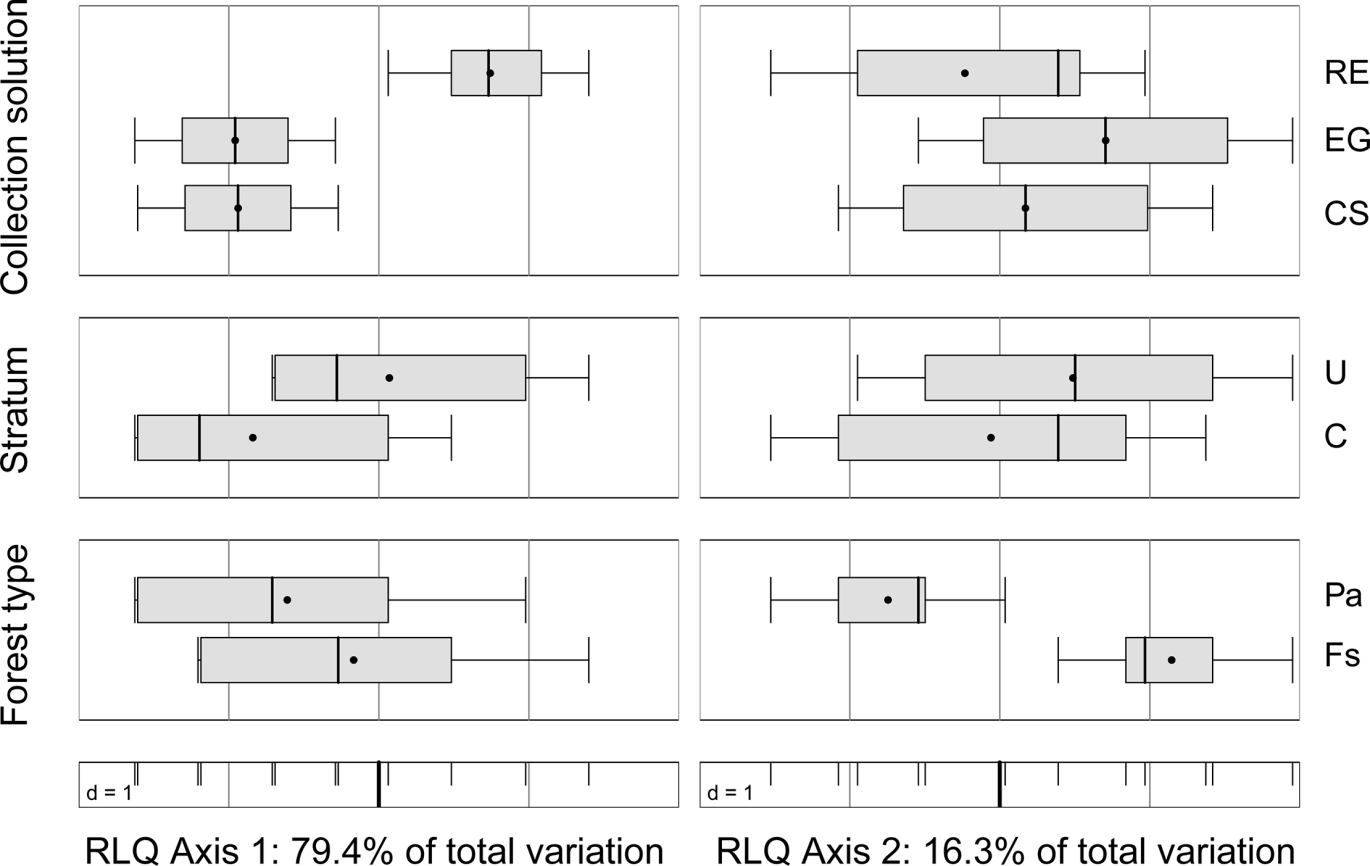
First and second ordination axis of the RLQ analysis. Position of beetle species at the average score. Horizontal lines are standard deviation of scores. Boxplot of the standardised scores of the environmental variables: Sampling solution (RE = Renner solution, EG = ethylene glycol, CS = copper sulphate); Stratum (U = understorey, C = canopy); Forest type (Pa = Spruce *Picea abies*, Fs = Beech *Fagus sylvatica*) on the first (left) and second (right) RLQ-axis. Species names and position along the first and second RLQ axis are given in Table A in S6 File.

Due to the low number of adult Hemiptera in the samples we restricted the analyses of community composition to Coleoptera. The changes in the community composition are illustrated by RLQ analysis. The first axis of the RLQ analysis explained 79.4% and the second axis 16.3% of the total variation. The RLQ analysis captured 92.7% and 81.5% of the total inertia of the R–L and the Q–L analyses on the first RLQ axis indicating that the environment–species relationship and the trait–species relationships are both very close in our dataset. Sampling solution had a major influence on the composition of the beetle assemblages along the first RLQ axis (Fig 3). The high explanatory value of the second RLQ axis on the other hand is due to the high significance of the factor "forest type".

**Fig 4:**
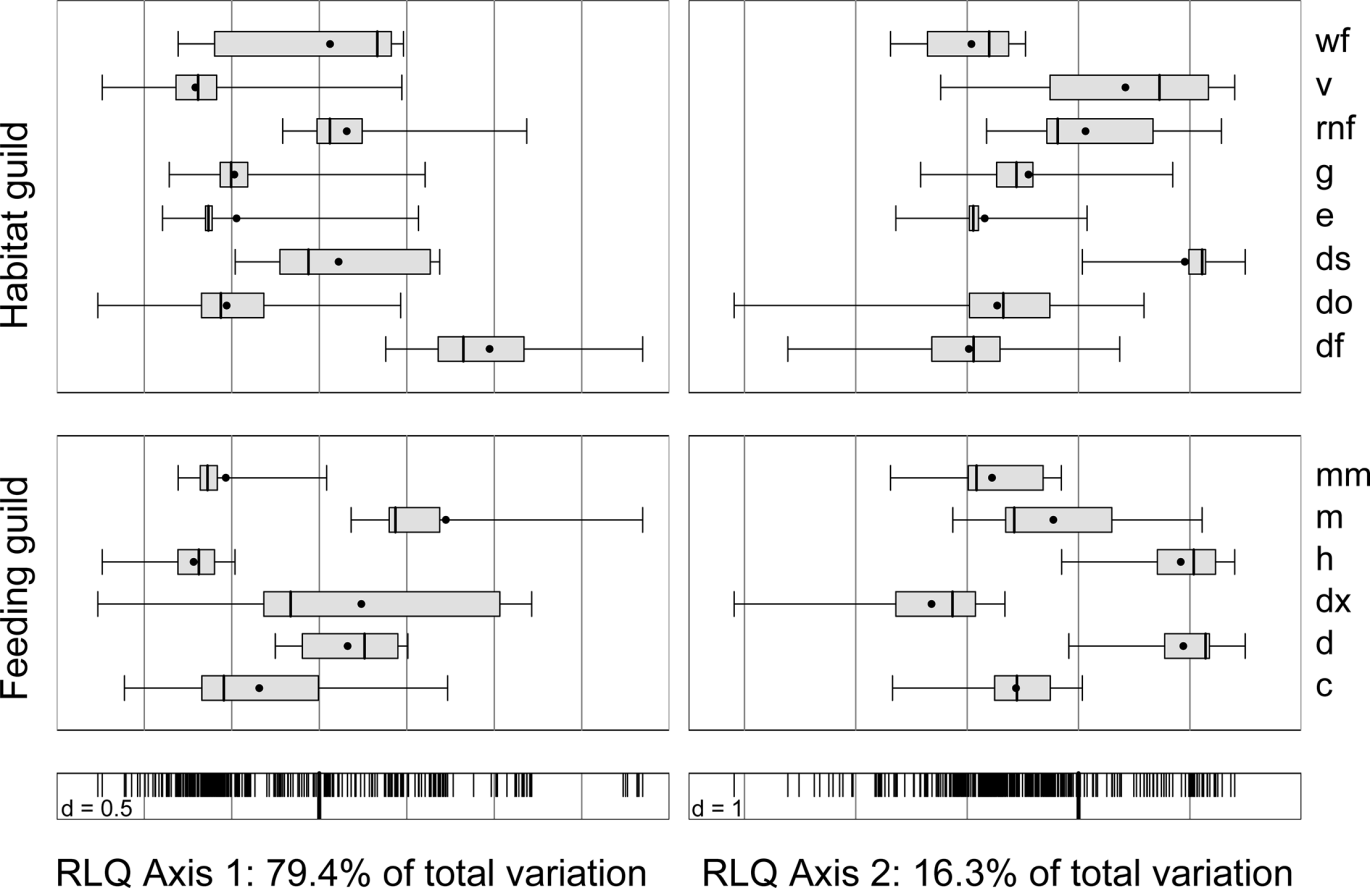
Distribution of Coleoptera trait categories along the first (left) and second (right) RLQ axis. The right part of the axis 1 is associated with the Renner solution (RE) and the understorey (U), the left with the other two sampling solution (EG: ethylene glycol, CS: copper sulphate) and the canopy (C); the right part of the axis 2 is associated with the beech forests (FS: *Fagus sylvatica*), the left with the spruce forests (FS: *Picea abies*) (see Fig 3). Horizontal boxplots display the weighted average position (points) of species trait categories in the Coleoptera community, including 25% and 75% quartiles and min and max values. Bottom dots with vertical lines are the weighted average positions of the species along the first RLQ-axis. See Table A in S6 File for species positions along the axes. Feeding guilds: c = carnivore, d = decomposer (excl. wood), dx = decomposer-wood, h = herbivore (excl. xylophage), m = mycetophagous-fungi, mm = mycetophagous-mould; Habitat guilds: df = fresh dead wood, do = old dead wood, ds = specific dead wood structures at living trees, e = eurytop, g = ground dweller, rnf = rotten substrate/nests/fungi (excl. wood), v = vegetation, wf = wood fungi.

The correlation between beetle traits and the RLQ axes showed that feeding guild (Correlation Ratios CR; axis 1: 0.33, axis 2: 0.16) and habitat preference (CR axis 1: 0.54, axis 2: 0.06) but not body size (CR axis 1: 0.06, axis 2: 0.06) were well represented along the gradient of the two axes. Fresh dead wood dwellers, among those mainly mycetophagous ambrosia beetles, were pronounced in traps with Renner solution (Fig 4; Table A in S6 File). While wood-decomposers were more pronounced in spruce forests, other decomposers and herbivores were more important in beech forests (Fig 4). In a fourth corner analyses, however, only the relationship between habitat guild and sampling solution was significant (Table A in S6 File). A complete list of all sampled species is given in Table A in S7 File.

### Species identification through DNA barcoding

In total, 65 individuals (of 150 total) were successfully identified using genetic barcoding.

**Fig 5:**
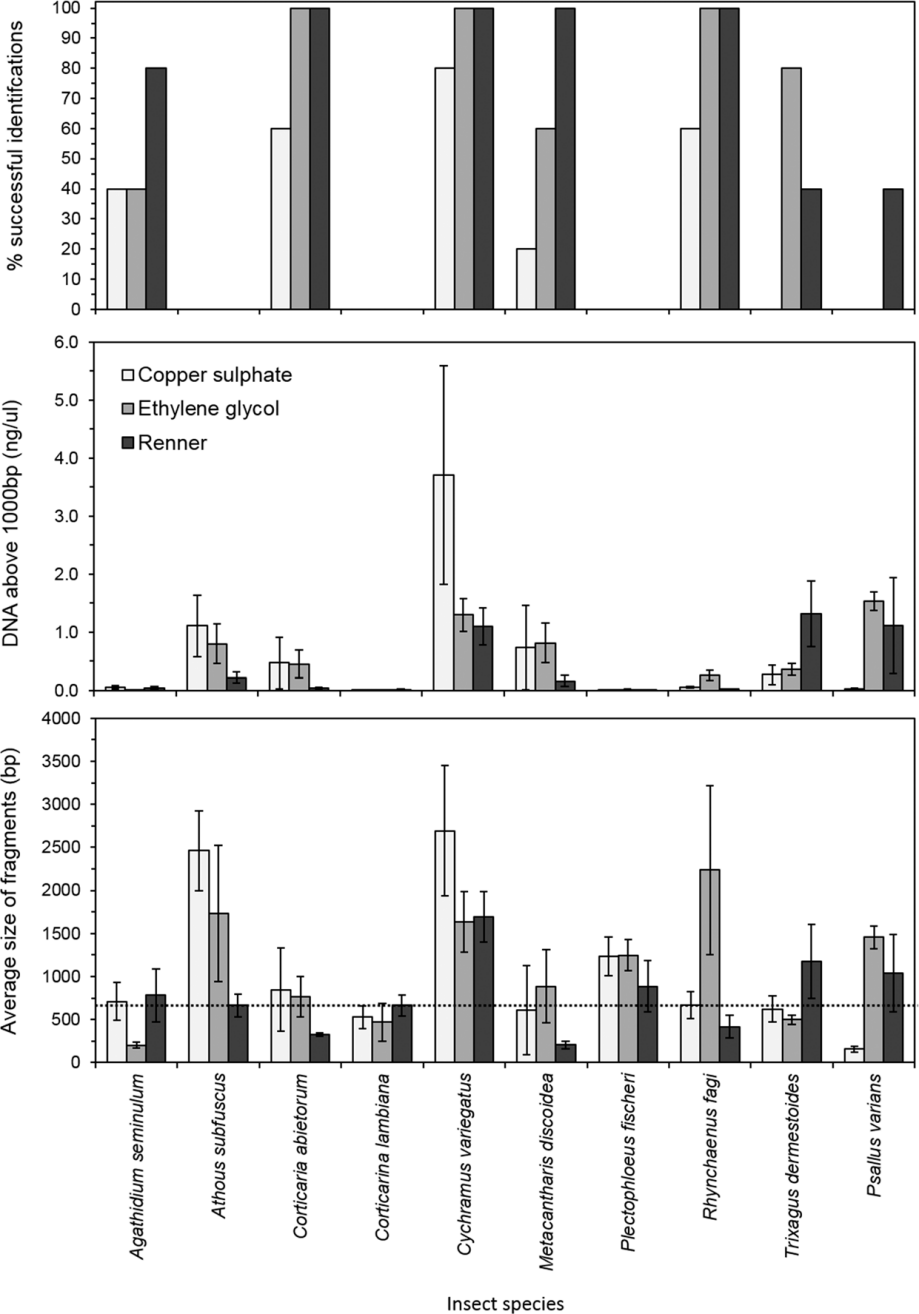
DNA yield and quality of studied species. The percentage of samples with successful identification of the genetic barcode (top), the concentration of DNA above 1000bp (ng/μl) and average length of recovered DNA fragments (bp), with the dotted line showing the 658bp fragment length required (bottom), across the solutions and species tested. DNA yield and fragment length was measured by a Fragment Analyzer. Error bars ±1SE.

Three species did not produce any successful barcode sequences (*A*. *subfuscus*, *C*. *lambiana* and *P*. *fischeri*), whereas almost all individuals in another three species were correctly identified from the barcoding sequencing (*C*. *abietorum*, *C*. *variegatus* and *R*. *fagi*) (Fig 5). The number of successful identifications was therefore strongly species-specific (GLM binomial: F_9,149_ = 23.65, P<0.001) and was further influenced by sampling solution (F_2,149_ = 9.41, df = 2, P<0.001), with copper sulphate samples producing fewer successful identification than ethylene glycol (t = 2.87, P = 0.004) or Renner (t = 3.88, P<0.001) (Fig 5). There was no significant interaction between species and solution on barcoding success.

DNA yield varied across the species (average 15.30–634.99 ng/mg; F_9,120_ = 15.29, P<0.001) and was dependent on the sampling solution (interaction: F_18,120_ = 1.71, P = 0.045; Table 3). We aimed to sequence a 658 bp fragment of the CO1 gene, and we found that fragmentation of the DNA also explained variation in obtaining a successful sequence across the different species and solutions. The average fragment length of the DNA and the concentration of DNA above 1000bp were correlated (r = 0.666, P<0.001) and both were influenced by a significant species by solution interaction (fragment length: F_18,120_ = 3.12, P<0.001; concentration above 1000bp: F_18,120_ = 2.23, P = 0.005) (Fig 5). There was no evidence that higher DNA yield, in general, produced more successful sequence identifications (GLM binomial: Χ^2^ = 2.46, df = 1, P = 0.117), but samples with larger average fragment length and higher concentration of good DNA (above 1000bp) did lead to higher sequencing success (GLM binomial: length Χ^2^ = 6.48, df = 1, P = 0.011; concentration above 1000bp Χ^2^ = 14.12, df = 1, P<0.001). In particular, *P*. *fischeri* samples produced very low DNA yield (15.30 ± 4.5 ng/mg; Table 3) (posthoc: t = -2.27, P = 0.025) and, while the average length was around 1000bp the extremely low concentration of DNA led to no successful barcoding sequences being obtained from this species. However, many species-solution combinations had lower average fragment length than the required 658bp and yet successful sequences were obtained, indicating that while success rate was increased with better quality DNA this could not explain all the variation in the data. We calculated that samples with at least 3.1 ng/μl of DNA above 1000bp would lead to an 80% success rate of sequence identification (Fig A in S8 File).

**Table 3:** DNA yield recovered from all species across the sampling solutions. DNA yield was measured by using a Quantus™ Fluorometer (Promega).

	DNA yield (ng/mg)		
Species	Copper sulphate	Ethylene glycol	Renner
*Agathidium seminulum*	137.82 ± 66.91	937.89 ± 373.45	170.68 ± 77.25
*Athous subfuscus*	595.96 ± 67.49	375.45 ± 108.94	155.59 ± 48.70
*Corticaria abietorum*	113.72 ± 66.38	223.00 ± 114.23	25.48 ± 8.20
*Corticarina lambiana*	124.68 ± 32.73	212.78 ± 84.94	171.48 ± 97.11
*Cychramus variegatus*	263.77 ± 71.33	241.45 ± 16.36	280.17 ± 50.13
*Metacantharis discoidea*	453.40 ± 110.21	565.18 ± 213.51	541.51 ± 167.62
*Plectophloeus fischeri*	18.43 ± 9.29	10.50 ± 0.55	16.97 ± 10.78
*Rhynchaenus fagi*	221.02 ± 88.55	921.31 ± 514.25	113.21 ± 43.08
*Trixagus dermestoides*	225.68 ± 93.38	855.65 ± 203.19	823.64 ± 164.54
*Psallus varians*	749.45 ± 289.83	747.95 ± 114.68	722.40 ± 292.20

Notes: values are mean ± standard error

There was little association between sample condition and DNA yield (F_1,71_ = 0.90, P = 0.345) or average fragment length (F_1,71_ = 2.05, P = 0.157). But, lower DNA yield was to some extent associated with a lower pH of the solution, i.e. in copper sulphate samples (F_1,80_ = 3.19, P = 0.078), although the average fragment length was not affected (F_1,80_ = 0.67, P = 0.414). Further, of the successful identified sequences the obtained fragments after sequencing (and therefore the resulting consensus strands) were on average shorter when copper sulphate was used (630 bp) than ethylene glycol (650 bp) or Renner (646 bp). The quality of the DNA sequences obtained was categorized as ‘high’ for 77%, 75%, 75% of samples from copper sulphate, ethylene glycol and Renner, respectively. Thus, while the number of successful identifications and length of sequence were lower for copper sulphate samples, for those successful sequences obtained the sequence quality was not worse than for the other sampling solutions.

## Discussion

Our study showed that condition of samples as well as the composition of sampled arthropod communities clearly depends on the sampling solution. More importantly, however, we could show that effects of sampling solution strongly depend on the forest type, the vertical stratum, and also whether top or bottom jars were used. Samples were more mouldy and fragmented in top jars filled with copper sulphate solution and in bottom jars in general when compared to top jars filled with ethylene glycol and Renner solution. Community parameters in terms of orders were highly affected by forest type and vertical stratum, but less so by sampling solution. Species richness and community composition, however, differed greatly among sampling solutions. Renner solution had either a highly attractant (beetles) or repellent (true bugs) effect on species when comparing to the other solutions. The change in community composition by sampling solution was mainly related to ambrosia beetles which colonize fresh dead wood and were most likely attracted by ethanol containing Renner solution. We found that DNA barcoding was highly successful for three species, very unsuccessful for three species and the other four species tested produced variable results. Overall, samples collected in copper sulphate showed lower barcoding success than for the other two sampling solutions, which was not directly related to sample condition or reduced DNA yield although the low pH of copper sulphate did influence DNA yield to some degree. Possible alternative underlying mechanisms are discussed below.

### Sample condition

The difference in observed sample conditions was not affected by pH, although pH differed between solutions and changed under field conditions. The generally more mouldy and fragmented insects of samples from the bottom jars might be caused by a negative rainfall-related dilution effect. Samples from ethylene glycol were generally less mouldy and fragmented than the other solutions, particularly in beech forests, which indicates that this solution serves as the best alternative under extreme rainfall-caused sample dilution. The sample conditions in the top jars, where solution was not diluted, was generally better for ethylene glycol or Renner solution than in the bottom jar, but not so for copper sulphate. The better sample preservation of ethylene glycol and Renner solution when compared with copper sulphate is in line with the predictions on the basis of pitfall traps [5,61], where a roof is often used as protection to rainwater. Schmidt et al. [36] conclude from their study on spiders and beetles in pitfall traps that ethylene glycol has even better conservation attributes than any solution containing ethanol. In particular in soft-bodied species, i.e. spiders, they found a softening of the cuticle due to decomposition and/or chemical processes when using water, ethanol-water or ethanol-glycerine. The incidence of mould on species sampled in top jars filled with copper sulphate is also in line with the observations of samples collected in similar jars attached to stem eclectors in spruce forests [61]. This is, however, somehow surprising as copper-containing fungicides including copper sulphate are widely used in agriculture [62–64]. Possibly the high humidity in the top jars resulting from water evaporation allows moulding at the surface of the copper-sulphate solution. The differences in conditions, however seemed not to change attractiveness of sampling solution in our study, contrasting to other studies that showed e.g. an attraction of Diptera by decay-induced volatiles [36].

The difference in sample conditions might also be an indication of suitability for subsequent morphological [36,65] or genetic analyses. Effects might depend on the taxonomic group as different cuticle consistency of e.g. soft bodied spiders vs. hard bodied beetles might influence moulding and DNA fragmentation [66]. For example, Dillon et al. [67] showed high DNA quality of Hymenoptera and Stoeckle et al. [5] of beetles preserved in ethylene glycol, while A’Hara et al. [68] found substantial degradation of DNA after preserving spiders in ethylene glycol for 3 weeks at room temperature. Therefore there is a need of comprehensive studies analysing DNA quality of species sampled by different sampling solutions in a multi-taxa approach.

### Effects of sampling solutions on insect communities

Analyses on order level did not reveal differences among sampling solutions. Thus, order-level analyses based on flight-interception traps are likely to be comparable among studies using our sampling solutions. Other studies based on pitfall traps, however, found significant differences in the sampled number of individuals among sampling solutions [36,61]. Beside the above mentioned decay-induced attraction of Diptera to Renner solution due to ethanol evaporation [36], an attraction of flies (Brachycera), snails and slugs (Gastropoda), and wasps (Hymenoptera: Vespidae) to ethylene glycol was observed [61]. Copper sulphate is described as being least attractant [61]. The lower numbers of spider individuals and beetle genera sampled in ethanol-glycerine and brine than in ethylene glycol, ethanol-water and water in the study of Schmidt et al. [36], suggests that glycerine might have deterrent effects to some groups. Thus comparison of studies based on pitfall traps using different sampling solution might be more critical.

Several studies suggest that type of preservative used can have substantial effects on abundance and species composition of carabids collected in pitfall traps [69–71], although this seems not to be a general rule [61]. These studies report of an attraction effect of ethylene glycol. In our study no attraction of either ethylene glycol of copper sulphate when compared to Renner solution was found. Renner solution, however, had a significant attracting effect to fresh dead wood colonizers. It has been shown, that ethanol is released in decaying wood, probably by microorganisms, and that this attracts bark beetle species, among others [72–75]. Thus we are confident that the observed differences between sampling solutions are due to attraction by Renner solution rather than repellent effect of the other solutions. We also found an effect of Renner on Heteroptera, which means they are either repelled by Renner or attracted by the other solutions, but there is a lack of supporting evidence in the literature to determine the more likely mechanism. This suggests that communities sampled by Renner solution cannot be compared to those sampled with other solutions, but comparisons between communities sampled by copper sulphate and ethylene glycol seem to be less biased.

### Interaction between sampling solution and forest type/stratum

The abundance of Diptera was higher in the canopy than in the understory, a pattern which was found only in part in previous studies (e.g., [76]) and might highly depend on the structure and heterogeneity of the forest [77]. While Diptera were more abundant in spruce compared to beech forests, the opposite was found for Thysanoptera. This might be explained by more humid conditions in spruce forests and tree species specificities. The higher species richness of beetles in the understory compared to the canopy and in spruce compared to beech forests is in line with other studies in Central Europe [78–80]. Saproxylic beetles comprise 30% of all beetle species in forests [81]. The higher availability of dead wood resources for saproxylic beetles in the understory than in the canopy and in spruce compared to beech forests in our study might explain this pattern. The dead wood distribution is a general pattern found in commercial forests of Europe [17,82,83]. In Hemiptera no effect of forest type and stratum was observed. Also, other studies did not find a difference in species richness between spruce and beech forests [84] and vertical stratification depended on forest type and forest openness [85].

Furthermore, we found that effects of sampling solution highly depended on the forest type and the vertical stratum. In the canopy only a marginal attracting effect of Renner solution was observed, and exclusively in beech forests. In the understory, the attractive effect of Renner solution was highly significant and more pronounced in spruce forests. This is mainly an effect of the higher abundance and species richness of fresh wood dwellers in the understory compared to the canopy and in spruce forests compared to beech forests [78]. Due to these interaction effects comparisons among sampling solutions (Renner vs. ethylene glycol / copper sulphate) seem to be less biased when focusing on canopy compared to understory communities. Nevertheless, such comparisons should be done with caution.

### Species identification through DNA barcoding

The striking contrast between species for which almost all samples provided a successful sequence for identification and those that produced none highlights the strong species-specific effect of using barcoding as a tool for species identification. We found that samples generally had lower barcoding success when they were sampled in copper sulphate solution, which is consistent with the effects on sampling condition previously discussed. However, while copper sulphate solution reduced the number of successful sequences, it produced similar yields of DNA as from the Renner solution. This suggests copper sulphate may lead to an increased rate of DNA degradation, leading to no suitable COI gene fragment from which to amplify the sequence and we did obtain shorter sequence lengths obtained from copper sulphate samples in our successful identifications. However, the overall average length of fragments in samples from copper sulphate were not substantially lower than from the other solutions, but there was much more variation both among and within the different species for copper sulphate collected samples. The mutagenic effect of copper ions as well as its supporting effect of DNA breaks has been frequently discussed [86–88]. Copper might negatively affect DNA synthesis and leads to single base substitutions [89], potentially influencing the amplification of sequences during PCR. We also found evidence that the low pH of copper sulphate may also lead to reduced DNA yield, and it is known that neutral or alkaline pH is preferable for limiting DNA degradation [90]; however, pH did not have a significant effect on identification success of the samples.

By using universal primers, such as LCO1490 and C1-N-2191, we assume the primer binding sites are conserved across the species studied [49,50]. However, albeit unlikely, changes in the primer binding sites may explain why *A*. *subfuscus* produced no sequence results despite seemingly sufficient DNA. The other two species with no successful amplifications were shown to have very low concentrations of good (above 1000bp) DNA and this therefore explains the failure of sequencing in these species. From the data, we calculated that a concentration of 3.1 ng/μl of DNA above 1000bp would be required to achieve an overall 80% success rate. We used only one set of universal DNA barcoding primers with an expected sequence length of 658 bp, to assess the potential for a single method (PCR conditions and primers) for identifying insects (specifically Coleoptera) in field samples. In a large biodiversity experiment using genetic analysis tools, the choice of sampling solution and species of interest will therefore have a large impact on the success of the work. Due to the species-specific success, it would be advisable to test multiple primer pairs on the species of interest when starting a new study. This is also important to avoid biasing the scientific research towards only those species that consistently produce good DNA for analyses.

We tested only the three most commonly used solutions in current biodiversity studies. Recently, Pokluda et al. [31] recommended to use 2% SDS and 100mM EDTA as a cheap, stable and easily transportable alternative to ethanol for preserving specimens and their DNA collected in the field. Its attracting effect has, however, not been tested and while it may be good for DNA it is unknown if community biases (as we found for Renner) might occur.

## Conclusions and Recommendation

In biodiversity studies many different properties of sampling solutions have to be considered; costs, toxicity, evaporation, attractiveness to arthropod taxa, and good preservation properties for subsequent morphological and genetic analyses. Based on our results and by considering previous studies we provide the following recommendations:

1. To obtain optimally preserved insects we suggest using ethylene glycol instead of Renner solution or copper sulphate as this solution had better preserved samples in all tested microclimatic situations. Propylene glycol might be used as a less toxic, but even more expensive alternative as it showed no different attraction compared to ethylene glycol in previous studies [91].
2. When decisions on sampling solutions are financially restricted and morphological identification is targeted, copper sulphate is suggested to be a suitable alternative because it costs only 7% of the price of ethylene glycol. By reducing sampling intervals, moulding of copper sulphate samples most likely could be minimised.
3. Meta analyses of data sampled with flight-interception traps using ethylene glycol or copper sulphate are assumed to be insignificantly biased, because we found–in contrast to pitfall trap studies- no differences in abundance, species richness or community composition between samples. Comparisons with samples caught by Renner solution should, however, be critically questioned.
4. When aiming at subsequent DNA analyses ethanol has mostly been used in the past. Because evaporation might decrease the preserving property of ethanol under field conditions, ethylene or propylene glycol might be an alternative [30,69,92]. We find that samples from ethylene glycol and Renner perform similarly in producing correct sequences for species identification. Despite copper sulphate producing fewer successful sequences, we still achieved up to 80% success rate dependent on species. Due to the potential bias towards or against certain species by the Renner sampling solution we suggest ethylene glycol as the optimal sampling solution when genetic analysis tools are to be used in the study.

## Supporting Information

S1 File Details on Experimental design. **Fig A** shows the location of studied trees within the Wippenhauser forest near Freising, Germany.
(PDF)

S2 File Details on sample quality measure. **Table A** gives an overview on the criteria that were used to classify our samples in terms of quality for subsequent morphological species identification.
(PDF)

S3 File Results of preliminary study (older samples from 2008–2011). **Fig A** shows the mean DNA yield (ng/g) across the different sampling solutions and extraction methods, and percentage of successfully identified individuals across species and extraction method. **Table A** gives a summary of data from the preliminary study comparing DNA and barcoding success from seven species, using two extraction methods from four sampling solutions.
(PDF)

S4 File Detailed results on order level. **Table A** gives the number of individuals collected in the Beech and Spruce understory and canopy traps, by order.
(PDF)

S5 File Detailed results regarding sample condition. **Table A** gives a summary of the results for sample condition. **Fig A** shows dilution curves of the sampling solutions.
(PDF)

S6 File Detailed results on insect community measures. **Fig A** shows the species richness of Coleoptera samples collected, across all treatments. **Table A** shows the species positions along the first and second RLQ axis (sorted by RLQ1) referring to Figs 2 and 3 of the main manuscript.
(PDF)

S7 File Overview on sampled species. **Table A** gives a list of species including abundance sampled over seven weeks between May and July 2013, separated by forest type, stratum and top and bottom jars of flight-interception traps.
(PDF)

S8 File Additional information on DNA barcoding. **Fig A** shows the probability of successful ID of a sample as a function of the concentration of good DNA in the sample. **Fig B** shows the probability of successful ID of a sample as a function of the % of good DNA in the sample.
(PDF)
