# Supplementary material for: Searching for the Optimal Sampling Solution: Variation in Invertebrate Communities, Sample Condition and DNA Quality
Source: PLoS One. 2016 Feb 3;11(2):e0148247. doi: 10.1371/journal.pone.0148247 (PMC4740435; doi:10.1371/journal.pone.0148247)
Supplement: S1 File — Fig A shows the location of studied trees within the Wippenhauser forest near Freising, Germany. (PDF) [file pone.0148247.s001.pdf]

Supplementary S1: Details on Experimental design

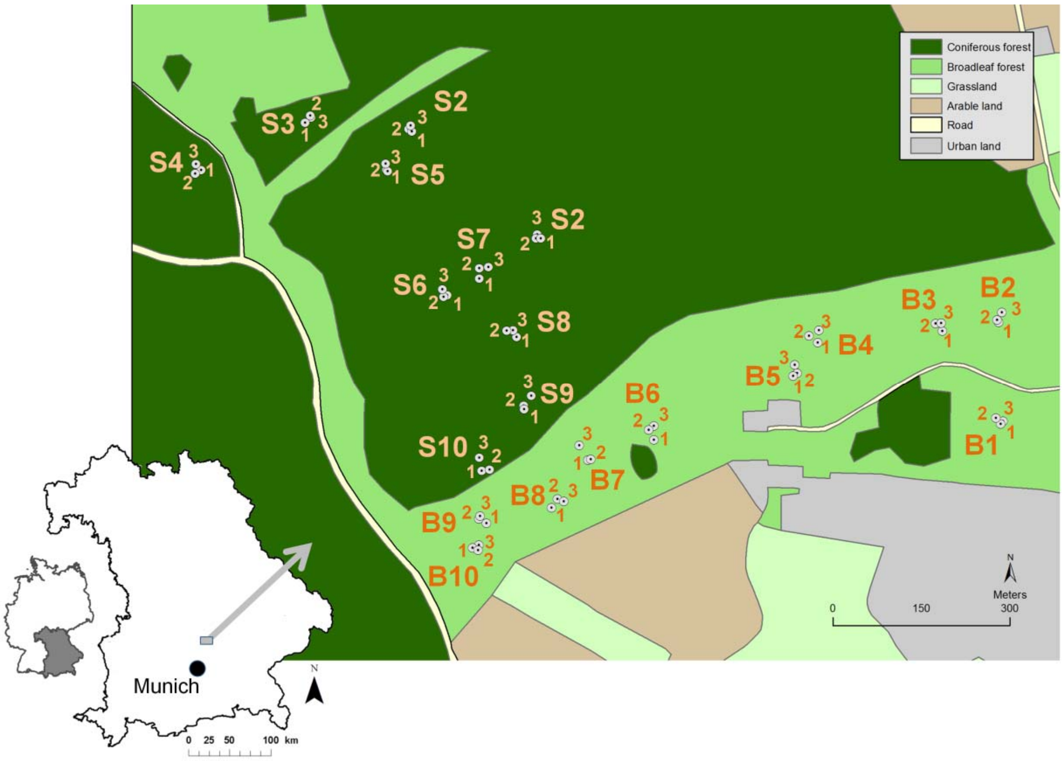

Figure A: Map showing the location of studied trees within the Wippenhauser forest near Freising, Germany. The experimental blocks are indicated by big letters (B = Beech *Fagus sylvatica*, S = Spruce *Picea abies*). Each block consisted of one forest plot with three trees, one each for the three sampling solutions (1 = copper sulphate ($CuSO_4$ 3%), 2 = ethylene glycol (50%), 3 = Renner solution (ethanol 40% / glycerine 25% / water 35%). The inset shows the location of the experimental sites within Bavaria within Germany.
