# Supplementary material for: Searching for the Optimal Sampling Solution: Variation in Invertebrate Communities, Sample Condition and DNA Quality
Source: PLoS One. 2016 Feb 3;11(2):e0148247. doi: 10.1371/journal.pone.0148247 (PMC4740435; doi:10.1371/journal.pone.0148247)
Supplement: S2 File — Table A gives an overview on the criteria that were used to classify our samples in terms of quality for subsequent morphological species identification. (PDF) [file pone.0148247.s002.pdf]

Supplementary S2: Details on the measure of quality for morphological species determination

To evaluate the quality for morphological species determination we focused on the samples of June based on two criteria, mould in the samples and completeness of insects (Table A). For each sample we assigned a value of 0.75 to optimally preserved samples showing no traces of mould and comprising exclusively complete insects. A value of 3.25 was assigned to samples in which insects were covered in a plug of mould and largely fragmented. For all other samples single values were assigned for both criteria according to table S2-1 and the mean of both values were used for further analyses. This resulted in a scale from 0.75 to 3.25 in steps of 0.25.

Table A: Overview on the criteria that were used to classify our samples in terms of quality for subsequent morphological species identification.

Value	Mould	Insect completeness
0.75	no mould at all and all insects complete	
1	small traces of mould, but specimens without mould	single body parts missing in less than 5% of the specimens
1.5	medium traces of mould, but specimens without mould	body parts are missing from more than 10% of the specimens
2	bigger traces of mould, but specimens without mould	body parts are missing from more than 50% of the specimens
2.5	bigger traces of mould and specimens slightly covered by mould	body parts are missing from more than 90% of the specimens
3	bigger traces of mould and specimens conspicuously covered by mould	extremities are missing from all specimens
3.25	insects in a plug of mould and largely fragmented	
