# Supplementary material for: Searching for the Optimal Sampling Solution: Variation in Invertebrate Communities, Sample Condition and DNA Quality
Source: PLoS One. 2016 Feb 3;11(2):e0148247. doi: 10.1371/journal.pone.0148247 (PMC4740435; doi:10.1371/journal.pone.0148247)
Supplement: S3 File — Fig A shows the mean DNA yield (ng/g) across the different sampling solutions and extraction methods, and percentage of successfully identified individuals across species and extraction method. Table A gives a summary of data from the preliminary study comparing DNA and barcoding success from seven species, using two extraction methods from four sampling solutions. (PDF) [file pone.0148247.s003.pdf]

**Supplementary S3: Results of preliminary study (older samples from 2008-2011)**

From the preliminary study, we recovered correct barcoding sequences from 37/138 Salting-Out [1] and 26/137 Phenol-Chloroform extracted samples. Identification success was dependent on both the species and extraction method (GLM quasibinomial: $F_{6,239}=2.69$, $P=0.015$) and also between species and the sampling solution (GLM quasibinomial: $F_{18,245}=3.60$, $P<0.001$). One species (*Anobium costatum*) produced no positive identifications from the sequencing data, whereas another (*Taphrorychus bicolor*) produced 29/40 correct identifications, with the success in this species dependent on the sampling solution. In general, using Ethanol as the sampling solution and extracted samples using the Salting-Out method recovered greater DNA yield and produced more positive sequence identifications (Fig A, Table A). Yield was measured on a Nanodrop 100 in the preliminary study, as opposed to a Quantus Fluorometer used in the main study.

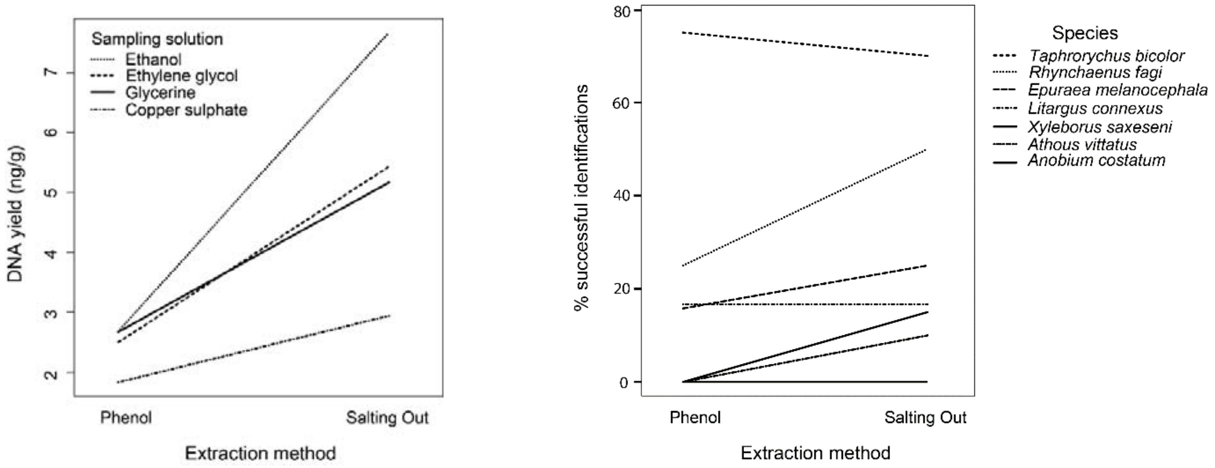


**Figure A:** Mean DNA yield (ng/g) across the different sampling solutions and extraction methods, and percentage of successfully identified individuals across species and extraction method.

**Table A:** Summary of data from the preliminary study comparing DNA and barcoding success from seven species, using two extraction methods from four sampling solutions.

| **Species** | **Solution** | **Extraction method** | **Mean DNA yield (ng/g)** | **ID success** | **No. samples** |
|---|---|---|---|---|---|
| *Anobium costatum* | Copper sulphate | Salting Out | 7.51 | 0 | 5 |
| *Anobium costatum* | Copper sulphate | Phenol | 2.51 | 0 | 5 |
| *Anobium costatum* | Ethanol | Salting Out | 7.04 | 0 | 5 |
| *Anobium costatum* | Ethanol | Phenol | 2.26 | 0 | 5 |
| *Anobium costatum* | Ethylene glycol | Salting Out | 5.38 | 0 | 5 |
| *Anobium costatum* | Ethylene glycol | Phenol | 3.39 | 0 | 5 |
| *Anobium costatum* | Glycerine | Salting Out | 1.65 | 0 | 5 |
| *Anobium costatum* | Glycerine | Phenol | 1.58 | 0 | 5 |
| *Athous vittatus* | Copper sulphate | Salting Out | 0.41 | 0 | 5 |
| *Athous vittatus* | Copper sulphate | Phenol | 1.70 | 0 | 5 |
| *Athous vittatus* | Ethanol | Salting Out | 1.60 | 2 | 5 |
| *Athous vittatus* | Ethanol | Phenol | 0.35 | 0 | 5 |
| *Athous vittatus* | Ethylene glycol | Salting Out | 0.45 | 0 | 5 |
| *Athous vittatus* | Ethylene glycol | Phenol | 1.23 | 0 | 5 |
| *Athous vittatus* | Glycerine | Salting Out | 3.13 | 0 | 5 |
| *Athous vittatus* | Glycerine | Phenol | 0.93 | 0 | 5 |
| *Epuraea melanocephala* | Copper sulphate | Salting Out | 2.62 | 0 | 5 |
| *Epuraea melanocephala* | Copper sulphate | Phenol | 0.58 | 0 | 5 |
| *Epuraea melanocephala* | Ethanol | Salting Out | 10.49 | 3 | 5 |
| *Epuraea melanocephala* | Ethanol | Phenol | 2.72 | 2 | 5 |
| *Epuraea melanocephala* | Ethylene glycol | Salting Out | 2.74 | 2 | 5 |
| *Epuraea melanocephala* | Ethylene glycol | Phenol | 1.69 | 1 | 5 |
| *Epuraea melanocephala* | Glycerine | Salting Out | 3.56 | 0 | 5 |
| *Epuraea melanocephala* | Glycerine | Phenol | 1.06 | 0 | 5 |
| *Rhynchaenus fagi* | Copper sulphate | Salting Out | 4.63 | 2 | 5 |
| *Rhynchaenus fagi* | Copper sulphate | Phenol | 1.69 | 1 | 5 |
| *Rhynchaenus fagi* | Ethanol | Salting Out | 2.40 | 1 | 5 |
| *Rhynchaenus fagi* | Ethanol | Phenol | 0.82 | 1 | 5 |
| *Rhynchaenus fagi* | Ethylene glycol | Salting Out | 9.90 | 3 | 5 |
| *Rhynchaenus fagi* | Ethylene glycol | Phenol | 4.17 | 2 | 5 |
| *Rhynchaenus fagi* | Glycerine | Salting Out | 6.95 | 4 | 5 |
| *Rhynchaenus fagi* | Glycerine | Phenol | 1.50 | 1 | 5 |
| *Taphrorychus bicolor* | Copper sulphate | Salting Out | 0.57 | 0 | 5 |
| *Taphrorychus bicolor* | Copper sulphate | Phenol | 2.68 | 2 | 5 |
| *Taphrorychus bicolor* | Ethanol | Salting Out | 13.41 | 5 | 5 |
| *Taphrorychus bicolor* | Ethanol | Phenol | 4.20 | 5 | 5 |
| *Taphrorychus bicolor* | Ethylene glycol | Salting Out | 11.63 | 4 | 5 |
| *Taphrorychus bicolor* | Ethylene glycol | Phenol | 2.58 | 3 | 5 |
| *Taphrorychus bicolor* | Glycerine | Salting Out | 13.49 | 5 | 5 |
| *Taphrorychus bicolor* | Glycerine | Phenol | 5.35 | 5 | 5 |
| *Xyleborus saxeseni* | Copper sulphate | Salting Out | 3.49 | 0 | 5 |
| *Xyleborus saxeseni* | Copper sulphate | Phenol | 2.88 | 0 | 5 |
| *Xyleborus saxeseni* | Ethanol | Salting Out | 6.89 | 3 | 5 |
| *Xyleborus saxeseni* | Ethanol | Phenol | 2.77 | 0 | 5 |
| *Xyleborus saxeseni* | Ethylene glycol | Salting Out | 7.36 | 0 | 5 |
| *Xyleborus saxeseni* | Ethylene glycol | Phenol | 2.54 | 0 | 5 |
| *Xyleborus saxeseni* | Glycerine | Salting Out | 5.53 | 0 | 5 |
| *Xyleborus saxeseni* | Glycerine | Phenol | 2.71 | 0 | 5 |
| *Litargus connexus* | Copper sulphate | Salting Out | 1.00 | 1 | 4 |
| *Litargus connexus* | Copper sulphate | Phenol | 0.55 | 0 | 4 |
| *Litargus connexus* | Ethanol | Salting Out | 11.88 | 2 | 5 |
| *Litargus connexus* | Ethanol | Phenol | 5.65 | 2 | 5 |
| *Litargus connexus* | Ethylene glycol | Salting Out | 0.60 | 0 | 5 |
| *Litargus connexus* | Ethylene glycol | Phenol | 1.91 | 0 | 5 |
| *Litargus connexus* | Glycerine | Salting Out | 1.06 | 0 | 4 |
| *Litargus connexus* | Glycerine | Phenol | 5.92 | 1 | 4 |

**References**

1. Sunnucks P, Hales DF (1996) Numerous transposed sequences of mitochondrial cytochrome oxidase I-II in aphids of the genus Sitobion (Hemiptera: Aphididae). Molecular Biology and Evolution 13: 510-524.
