# Supplementary material for: Searching for the Optimal Sampling Solution: Variation in Invertebrate Communities, Sample Condition and DNA Quality
Source: PLoS One. 2016 Feb 3;11(2):e0148247. doi: 10.1371/journal.pone.0148247 (PMC4740435; doi:10.1371/journal.pone.0148247)
Supplement: S4 File — Table A gives the number of individuals collected in the Beech and Spruce understory and canopy traps, by order. (PDF) [file pone.0148247.s004.pdf]

Supplementary S4: Detailed results on order level

Table A: Number of individuals collected in the Beech and Spruce understory and canopy traps, by order.

	Beech						Spruce						
	Copper sulphate		Ethylene glycol		Renner		Copper sulphate		Ethylene glycol		Renner		
	Under story	Canopy	Under story	Canopy	Under story	Canopy	Under story	Canopy	Under story	Canopy	Under story	Canopy	Total
**Acari**	1068	44	398	54	926	44	264	89	178	125	4341	58	**7589**
**Araneae**	156	18	82	23	76	32	48	49	65	39	76	41	**705**
**Chilopoda**	0	0	0	2	0	0	1	0	0	1	1	0	**5**
**Coleoptera**	351	240	287	176	811	238	667	341	674	215	1218	214	**5432**
**Collembola**	678	67	337	89	330	60	442	186	1221	187	639	217	**4453**
**Dermaptera**	6	3	7	3	6	1	2	0	0	0	0	0	**28**
**Dictyoptera**	12	13	12	22	6	19	8	12	8	11	8	8	**139**
**Diplopoda**	0	1	3	0	4	0	1	0	0	0	5	0	**14**
**Diptera**	1314	1943	1414	4775	1173	2193	3438	3027	2191	4535	1975	12357	**40335**
**Ephemeroptera**	0	0	0	0	0	0	0	3	3	7	1	3	**17**
**Gastropoda**	12	3	14	0	6	6	2	0	1	0	0	0	**44**
**Hemiptera**	177	282	104	266	109	217	467	354	483	290	419	300	**3468**
**Hymenoptera**	130	70	110	74	78	113	201	63	149	46	161	57	**1252**
**Isopoda**	17	0	19	1	12	0	32	2	25	0	9	3	**120**
**Lepidoptera**	10	29	7	19	7	10	14	9	22	7	11	17	**162**
**Mecoptera**	0	0	1	2	0	0	0	23	3	20	1	20	**70**
**Megaloptera**	0	0	0	0	0	0	0	7	0	18	1	3	**29**
**Neuroptera**	1	3	0	1	1	1	3	1	2	24	1	1	**39**
**Odonata**	0	0	3	0	0	0	0	2	0	0	0	0	**5**
**Oligochaeta**	1	1	1	1	1	0	0	0	0	1	0	0	**6**
**Opiliones**	5	0	0	0	2	0	1	2	2	3	5	2	**22**
**Orthoptera**	0	1	0	0	1	0	2	0	0	0	20	0	**24**
**Plecoptera**	0	1	0	1	0	0	2	6	3	13	0	10	**36**
**Pseudoscorpiones**	0	0	2	0	1	0	1	0	0	0	0	6	**10**
**Psocoptera**	24	38	15	24	15	23	134	94	114	77	112	92	**762**
**Raphidioptera**	0	0	0	0	0	0	1	0	1	1	0	0	**3**
**Siphonaptera**	0	0	0	0	0	0	0	0	0	1	0	0	**1**
**Thysanoptera**	2067	2321	1500	1528	1479	1755	257	290	162	51	156	245	**11811**
**Trichoptera**	1	2	0	0	0	1	0	2	0	0	0	1	**7**
