# Supplementary material for: Searching for the Optimal Sampling Solution: Variation in Invertebrate Communities, Sample Condition and DNA Quality
Source: PLoS One. 2016 Feb 3;11(2):e0148247. doi: 10.1371/journal.pone.0148247 (PMC4740435; doi:10.1371/journal.pone.0148247)
Supplement: S5 File — Table A gives a summary of the results for sample condition. Fig A shows dilution curves of the sampling solutions. (PDF) [file pone.0148247.s005.pdf]

Supplementary S5: Detailed results regarding sample condition

Table A: Summary of the results for sample condition

	Sample Condition		
**Source**	df	F	P
Tree species	1,18	24.56	**<0.001**
Solution	2,36	26.55	**<0.001**
Trap type	1,54	0.41	0.523
Jar	1,110	33.97	**<0.001**
Tree species x solution	2,36	4.64	**0.016**
Tree species x trap type	1,54	0.01	0.985
Solution x trap type	2,54	0.59	0.560
Tree species x jar	1,110	0.92	0.340
Solution x jar	2,110	15.45	**<0.001**
Trap type x jar	2,110	8.92	**0.004**
Tree species x solution x trap type	2,54	4.69	**0.013**

All interactions were tested, ones not shown were not retained in the minimum adequate model. Significant (P<0.05) terms are highlighted in bold.

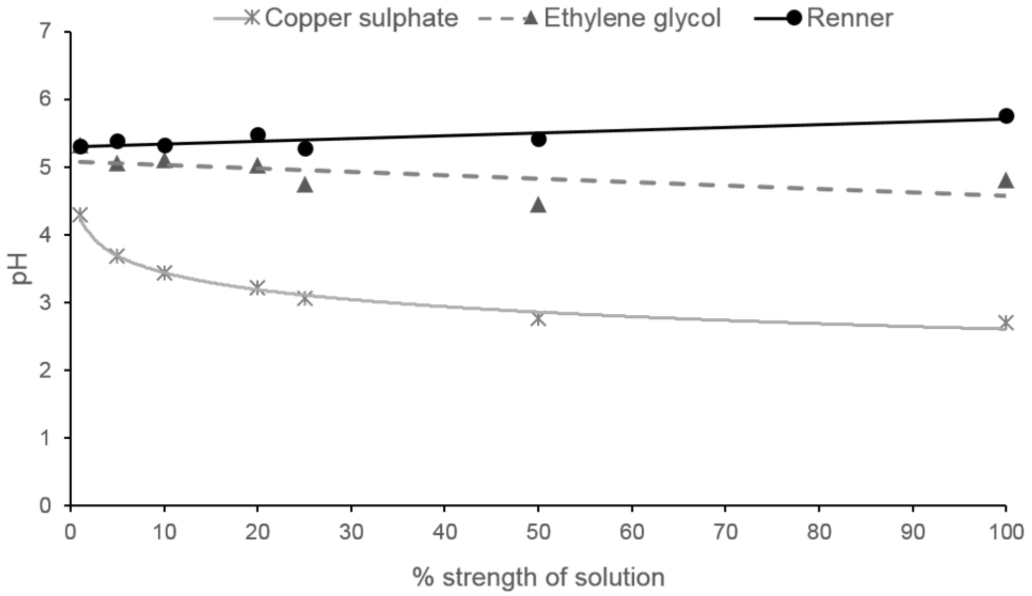

Figure A: Dilution curves of the sampling solutions. 100% strength means the same solution strength as used in the field. Samples were tested at 50, 25, 20, 10, 5 and 1% of the original solution strength. Only copper sulphate experienced a change in pH when the solution is diluted, particularly from 25% of the original. All dilutions were made with double-distilled water.
