# Supplementary material for: Searching for the Optimal Sampling Solution: Variation in Invertebrate Communities, Sample Condition and DNA Quality
Source: PLoS One. 2016 Feb 3;11(2):e0148247. doi: 10.1371/journal.pone.0148247 (PMC4740435; doi:10.1371/journal.pone.0148247)
Supplement: S6 File — Fig A shows the species richness of Coleoptera samples collected, across all treatments. Table A shows the species positions along the first and second RLQ axis (sorted by RLQ1) referring to Figs 2 and 3 of the main manuscript. (PDF) [file pone.0148247.s006.pdf]

Supplementary S6: Detailed results on insect community measures

Effect of sampling solution on species richness

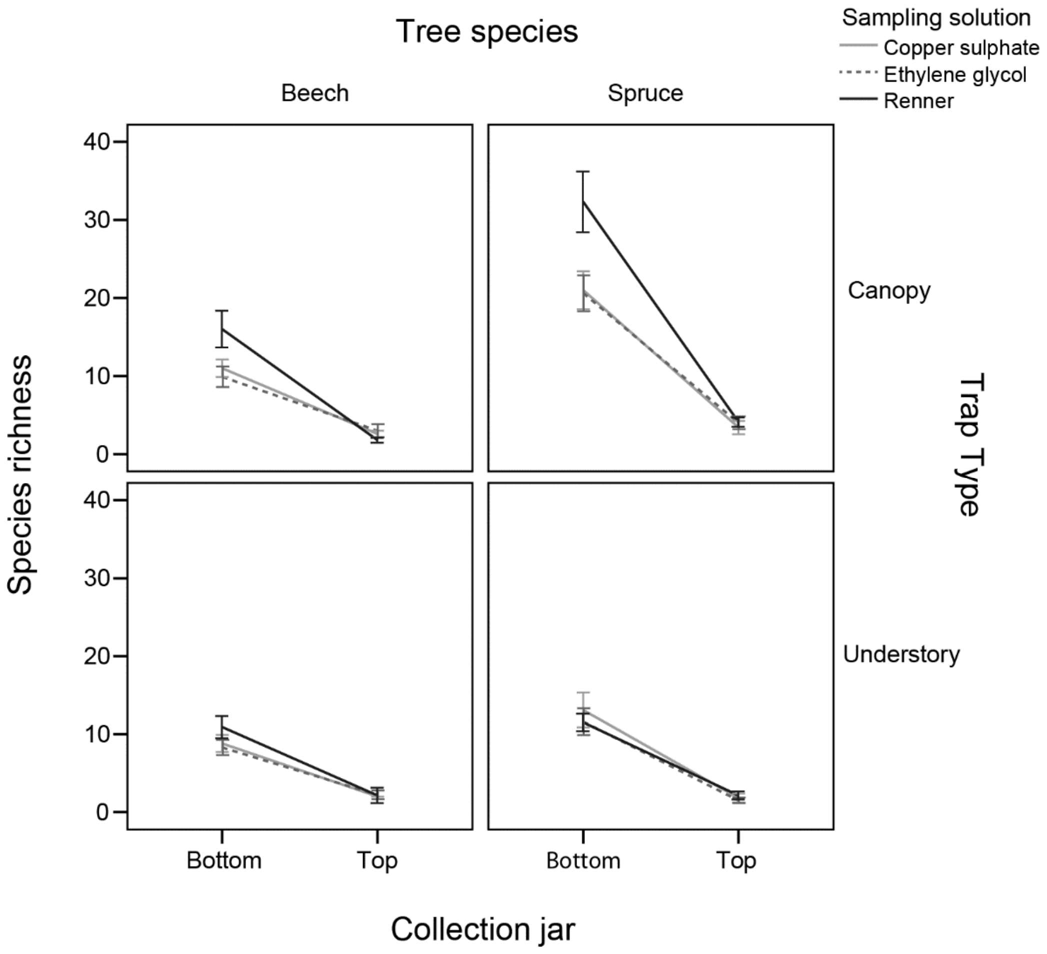

Figure A: The species richness of Coleoptera samples collected, across all treatments. A significant 3-way interaction (P=0.05) was found between tree species, sampling solution and jar for the species richness but not the diversity of Coleoptera. Error bars represent ±1SE.

Table A: Species positions along the first and second RLQ axis (sorted by RLQ1) referring to Figs 2 and 3 of the main manuscript. Additionally mean body size as well as feeding and habitat traits of species are given.

Order	Suborder	Family	SpeciesID	RLQ1	RLQ2	BodySize	Feeding	Habitat
Coleoptera	Polyphaga	Cerambycidae	*Oxymirus cursor*	-1.265	-3.093	23	dx	do
Coleoptera	Polyphaga	Elateridae	*Agriotes pilosellus*	-1.240	-0.152	14.75	h	v
Coleoptera	Polyphaga	Cantharidae	*Podabrus alpinus*	-1.113	-1.237	12.5	c	v
Coleoptera	Polyphaga	Elateridae	*Athous haemorrhoidalis*	-1.104	0.145	12.25	h	v
Coleoptera	Polyphaga	Cantharidae	*Cantharis pellucida*	-1.073	-1.149	11.75	c	v
Coleoptera	Polyphaga	Cleridae	*Opilo mollis*	-1.038	-1.217	10.75	c	do
Coleoptera	Polyphaga	Lycidae	*Dictyopterus aurora*	-1.025	-1.187	10.5	c	do
Coleoptera	Polyphaga	Elateridae	*Athous vittatus*	-0.996	0.382	10.25	h	v
Coleoptera	Polyphaga	Cantharidae	*Metacantharis discoidea*	-0.977	-0.941	10	c	v
Coleoptera	Polyphaga	Cantharidae	*Rhagonycha translucida*	-0.977	-0.941	10	c	v
Coleoptera	Polyphaga	Cantharidae	*Cantharis nigricans*	-0.950	-0.882	9.5	c	v
Coleoptera	Polyphaga	Elateridae	*Melanotus castanipes*	-0.939	-2.381	17	dx	do
Coleoptera	Polyphaga	Elateridae	*Athous subfuscus*	-0.936	0.513	9.15	h	v
Coleoptera	Polyphaga	Staphylinidae	*Quedius xanthopus*	-0.916	-0.950	8.5	c	do
Coleoptera	Polyphaga	Curculionidae	*Phyllobius pomaceus*	-0.901	0.590	8.5	h	v
Coleoptera	Polyphaga	Coccinellidae	*Anatis ocellata*	-0.896	-0.763	8.5	c	v
Coleoptera	Polyphaga	Staphylinidae	*Quedius mesomelinus*	-0.896	-1.640	9	c	e
Coleoptera	Polyphaga	Curculionidae	*Curculio pellitus*	-0.873	0.649	8	h	v
Coleoptera	Polyphaga	Curculionidae	*Curculio venosus*	-0.873	0.649	8	h	v
Coleoptera	Polyphaga	Coccinellidae	*Myzia oblongoguttata*	-0.869	-0.704	8	c	v
Coleoptera	Polyphaga	Staphylinidae	*Quedius maurus*	-0.861	-0.831	7.5	c	do
Coleoptera	Polyphaga	Staphylinidae	*Philonthus cognatus*	-0.857	-1.417	9.75	c	g
Coleoptera	Polyphaga	Curculionidae	*Otiorhynchus singularis*	-0.846	0.709	7.5	h	v
Coleoptera	Polyphaga	Staphylinidae	*Atrecus affinis*	-0.821	-0.742	6.75	c	do
Coleoptera	Polyphaga	Elateridae	*Agriotes acuminatus*	-0.819	0.768	7	h	v
Coleoptera	Polyphaga	Curculionidae	*Phyllobius arborator*	-0.819	0.768	7	h	v
Coleoptera	Polyphaga	Elateridae	*Ampedus sanguineus*	-0.817	-2.114	14.75	dx	do
Coleoptera	Polyphaga	Cantharidae	*Cantharis decipiens*	-0.814	-0.585	7	c	v
Coleoptera	Polyphaga	Coccinellidae	*Harmonia axyridis*	-0.814	-0.585	7	c	v
Coleoptera	Adephaga	Carabidae	*Amara ovata*	-0.808	0.054	8.75	h	g
Coleoptera	Adephaga	Carabidae	*Amara similata*	-0.808	0.054	8.75	h	g
Coleoptera	Polyphaga	Cryptophagidae	*Micrambe abietis*	-0.807	-1.688	2.3	mm	wf
Coleoptera	Polyphaga	Elateridae	*Kibunea minutus*	-0.805	0.797	6.75	h	v
Coleoptera	Polyphaga	Elateridae	*Dalopius marginatus*	-0.801	-0.555	6.75	c	v
Coleoptera	Polyphaga	Curculionidae	*Polydrusus impar*	-0.797	0.815	6.6	h	v
Coleoptera	Polyphaga	Cryptophagidae	*Atomaria procerula*	-0.796	-1.664	2.1	mm	wf
Coleoptera	Polyphaga	Latridiidae	*Corticaria abietorum*	-0.790	-1.652	2	mm	wf
Coleoptera	Polyphaga	Latridiidae	*Stephostethus rugicollis*	-0.779	-1.628	1.8	mm	wf

Coleoptera	Polyphaga	Tenebrionidae	*Stenomax aeneus*	-0.776	-2.025	14	dx	do
Coleoptera	Adephaga	Carabidae	*Dromius agilis*	-0.765	-0.478	6.1	c	v
Coleoptera	Polyphaga	Cantharidae	*Rhagonycha lignosa*	-0.760	-0.466	6	c	v
Coleoptera	Polyphaga	Latridiidae	*Corticarina lambiana*	-0.755	-1.575	1.35	mm	wf
Coleoptera	Polyphaga	Melyridae	*Dasytes caeruleus*	-0.753	-0.594	5.5	c	do
Coleoptera	Polyphaga	Curculionidae	*Curculio glandium*	-0.751	0.916	5.75	h	v
Coleoptera	Polyphaga	Staphylinidae	*Bolitochara obliqua*	-0.742	-1.670	3.6	c	wf
Coleoptera	Polyphaga	Omalisidae	*Omalisus fontisbellaquaei*	-0.735	-1.151	7.5	c	g
Coleoptera	Polyphaga	Cantharidae	*Rhagonycha testacea*	-0.733	-0.407	5.5	c	v
Coleoptera	Polyphaga	Staphylinidae	*Gabrius splendidulus*	-0.725	-0.535	5	c	do
Coleoptera	Polyphaga	Silvanidae	*Uleiota planata*	-0.725	-0.535	5	c	do
Coleoptera	Polyphaga	Elateridae	*Melanotus rufipes*	-0.721	-1.906	13	dx	do
Coleoptera	Polyphaga	Cantharidae	*Rhagonycha atra*	-0.719	-0.377	5.25	c	v
Coleoptera	Adephaga	Carabidae	*Amara plebeja*	-0.713	0.262	7	h	g
Coleoptera	Polyphaga	Curculionidae	*Otiorhynchus scaber*	-0.710	1.005	5	h	v
Coleoptera	Polyphaga	Curculionidae	*Polydrusus undatus*	-0.710	1.005	5	h	v
Coleoptera	Polyphaga	Cryptophagidae	*Cryptophagus dorsalis*	-0.708	-0.374	2.2	mm	do
Coleoptera	Polyphaga	Cantharidae	*Malthodes marginatus*	-0.698	-0.476	4.5	c	do
Coleoptera	Polyphaga	Cryptophagidae	*Atomaria ornata*	-0.697	-0.351	2	mm	do
Coleoptera	Polyphaga	Curculionidae	*Phyllobius argentatus*	-0.697	1.035	4.75	h	v
Coleoptera	Polyphaga	Curculionidae	*Strophosoma melanogrammum*	-0.697	1.035	4.75	h	v
Coleoptera	Polyphaga	Melyridae	*Aplocnemus nigricornis*	-0.693	-0.464	4.4	c	do
Coleoptera	Polyphaga	Cryptophagidae	*Cryptophagus pilosus*	-0.691	-1.070	2.75	mm	e
Coleoptera	Polyphaga	Cryptophagidae	*Atomaria turgida*	-0.689	-0.333	1.85	mm	do
Coleoptera	Adephaga	Carabidae	*Pterostichus vernalis*	-0.689	-1.050	6.65	c	g
Coleoptera	Polyphaga	Elateridae	*Agriotes pallidulus*	-0.688	1.053	4.6	h	v
Coleoptera	Polyphaga	Staphylinidae	*Atheta myrmecobia*	-0.677	-1.528	2.4	c	wf
Coleoptera	Polyphaga	Latridiidae	*Stephostethus angusticollis*	-0.677	-1.040	2.5	mm	e
Coleoptera	Polyphaga	Melyridae	*Dasytes plumbeus*	-0.674	-0.422	4.05	c	do
Coleoptera	Polyphaga	Byturidae	*Byturus ochraceus*	-0.672	1.088	4.3	h	v
Coleoptera	Polyphaga	Cryptophagidae	*Cryptophagus dentatus*	-0.672	-1.028	2.4	mm	e
Coleoptera	Polyphaga	Melyridae	*Dasytes aeratus*	-0.671	-0.416	4	c	do
Coleoptera	Polyphaga	Cantharidae	*Malthinus frontalis*	-0.671	-0.416	4	c	do
Coleoptera	Polyphaga	Cryptophagidae	*Cryptophagus scanicus*	-0.666	-1.016	2.3	mm	e
Coleoptera	Polyphaga	Coccinellidae	*Adalia decempunctata*	-0.665	-0.259	4.25	c	v
Coleoptera	Polyphaga	Coccinellidae	*Aphidecta obliterata*	-0.659	-0.247	4.15	c	v
Coleoptera	Polyphaga	Cimberidae	*Cimberis attelaboides*	-0.656	1.124	4	h	v
Coleoptera	Polyphaga	Chrysomelidae	*Oulema melanopus*	-0.656	1.124	4	h	v
Coleoptera	Polyphaga	Rhizophagidae	*Rhizophagus nitidulus*	-0.652	-0.375	3.65	c	do
Coleoptera	Polyphaga	Coccinellidae	*Exochomus quadripustulatus*	-0.651	-0.229	4	c	v
Coleoptera	Polyphaga	Latridiidae	*Cartodere nodifer*	-0.650	-0.981	2	mm	e
Coleoptera	Polyphaga	Latridiidae	*Enicmus transversus*	-0.650	-0.981	2	mm	e
Coleoptera	Polyphaga	Ptiliidae	*Ptenidium laevigatum*	-0.649	-0.244	1.1	mm	do

Coleoptera	Polyphaga	Cryptophagidae	*Cryptophagus distinguendus*	-0.645	-0.969	1.9	mm	e
Coleoptera	Polyphaga	Cryptophagidae	*Atomaria analis*	-0.642	-0.963	1.85	mm	e
Coleoptera	Polyphaga	Curculionidae	*Barypeithes pellucidus*	-0.642	1.153	3.75	h	v
Coleoptera	Polyphaga	Ptiliidae	*Baeocrara variolosa*	-0.640	-0.226	0.95	mm	do
Coleoptera	Polyphaga	Latridiidae	*Enicmus histrio*	-0.636	-0.951	1.75	mm	e
Coleoptera	Polyphaga	Cryptophagidae	*Atomaria atricapilla*	-0.634	-0.945	1.7	mm	e
Coleoptera	Polyphaga	Byturidae	*Byturus tomentosus*	-0.634	1.171	3.6	h	v
Coleoptera	Polyphaga	Ptiliidae	*Pteryx suturalis*	-0.632	-0.208	0.8	mm	do
Coleoptera	Polyphaga	Cryptophagidae	*Atomaria fuscata*	-0.631	-0.939	1.65	mm	e
Coleoptera	Polyphaga	Latridiidae	*Corticarina fuscula*	-0.631	-0.939	1.65	mm	e
Coleoptera	Polyphaga	Elateridae	*Anostirus purpureus*	-0.626	-1.699	11.25	dx	do
Coleoptera	Polyphaga	Latridiidae	*Dienerella clathrata*	-0.626	-0.927	1.55	mm	e
Coleoptera	Polyphaga	Curculionidae	*Hypera nigrirostris*	-0.623	1.195	3.4	h	v
Coleoptera	Polyphaga	Staphylinidae	*Euryusa castanoptera*	-0.617	-0.298	3	c	do
Coleoptera	Polyphaga	Cantharidae	*Malthodes spathifer*	-0.617	-0.298	3	c	do
Coleoptera	Polyphaga	Bothrideridae	*Oxylaemus variolosus*	-0.617	-0.298	3	c	do
Coleoptera	Polyphaga	Latridiidae	*Corticarina similata*	-0.615	-0.904	1.35	mm	e
Coleoptera	Polyphaga	Staphylinidae	*Eusphalerum abdominale*	-0.615	1.213	3.25	h	v
Coleoptera	Polyphaga	Staphylinidae	*Leptusa pulchella*	-0.614	-0.292	2.95	c	do
Coleoptera	Polyphaga	Staphylinidae	*Philonthus albipes*	-0.613	-0.884	5.25	c	g
Coleoptera	Polyphaga	Latridiidae	*Cortinicara gibbosa*	-0.612	-0.898	1.3	mm	e
Coleoptera	Polyphaga	Curculionidae	*Rhynchaenus quercus*	-0.612	1.219	3.2	h	v
Coleoptera	Polyphaga	Curculionidae	*Stereonychus fraxini*	-0.607	1.230	3.1	h	v
Coleoptera	Polyphaga	Corylophidae	*Orthoperus mundus*	-0.601	-1.362	1	c	wf
Coleoptera	Polyphaga	Staphylinidae	*Leptusa fumida*	-0.600	-0.262	2.7	c	do
Coleoptera	Polyphaga	Curculionidae	*Anthonomus phyllocola*	-0.599	1.248	2.95	h	v
Coleoptera	Polyphaga	Elateridae	*Denticollis linearis*	-0.599	-1.639	10.75	dx	do
Coleoptera	Polyphaga	Silvanidae	*Silvanoprus fagi*	-0.598	-0.256	2.65	c	do
Coleoptera	Polyphaga	Corylophidae	*Orthoperus atomus*	-0.597	-1.354	0.935	c	wf
Coleoptera	Polyphaga	Coccinellidae	*Rhyzobius chrysomeloides*	-0.597	-0.110	3	c	v
Coleoptera	Polyphaga	Latridiidae	*Cartodere constricta*	-0.590	-0.711	2.35	mm	g
Coleoptera	Polyphaga	Elateridae	*Ampedus pomorum*	-0.585	-1.610	10.5	dx	do
Coleoptera	Polyphaga	Anthribidae	*Brachytarsus nebulosus*	-0.583	-0.081	2.75	c	v
Coleoptera	Polyphaga	Nitidulidae	*Meligethes denticulatus*	-0.582	1.284	2.65	h	v
Coleoptera	Polyphaga	Curculionidae	*Rhynchaenus fagi*	-0.580	1.290	2.6	h	v
Coleoptera	Polyphaga	Coccinellidae	*Scymnus abietis*	-0.575	-0.063	2.6	c	v
Coleoptera	Polyphaga	Staphylinidae	*Mycetoporus lepidus*	-0.572	-0.795	4.5	c	g
Coleoptera	Polyphaga	Rhynchitidae	*Caenorhinus germanicus*	-0.571	1.308	2.45	h	v
Coleoptera	Polyphaga	Nitidulidae	*Epuraea melanocephala*	-0.569	-0.051	2.5	c	v
Coleoptera	Polyphaga	Cerylonidae	*Cerylon histeroides*	-0.565	-0.185	2.05	c	do
Coleoptera	Polyphaga	Staphylinidae	*Leptusa norvegica*	-0.562	-0.179	2	c	do
Coleoptera	Polyphaga	Staphylinidae	*Phyllodrepa ioptera*	-0.562	-0.179	2	c	do
Coleoptera	Polyphaga	Pselaphidae	*Trichonyx sulcicollis*	-0.562	-0.179	2	c	do

Coleoptera	Polyphaga	Cryptophagidae	*Atomaria nigrirostris*	-0.560	-0.646	1.8	mm	g
Coleoptera	Polyphaga	Cerylonidae	*Cerylon ferrugineum*	-0.557	-0.167	1.9	c	do
Coleoptera	Polyphaga	Nitidulidae	*Epuraea unicolor*	-0.556	-0.898	2.75	c	e
Coleoptera	Polyphaga	Staphylinidae	*Gabrius coxalus*	-0.556	-0.759	4.2	c	g
Coleoptera	Polyphaga	Histeridae	*Paromalus flavicornis*	-0.554	-0.161	1.85	c	do
Coleoptera	Polyphaga	Staphylinidae	*Atheta incognita*	-0.553	-0.753	4.15	c	g
Coleoptera	Polyphaga	Staphylinidae	*Heterothops dissimilis*	-0.553	-0.753	4.15	c	g
Coleoptera	Polyphaga	Nitidulidae	*Meligethes aeneus*	-0.552	1.349	2.1	h	v
Coleoptera	Polyphaga	Nitidulidae	*Meligethes nigrescens*	-0.552	1.349	2.1	h	v
Coleoptera	Polyphaga	Nitidulidae	*Meligethes pedicularius*	-0.552	1.349	2.1	h	v
Coleoptera	Polyphaga	Staphylinidae	*Leptusa ruficollis*	-0.550	-0.010	2.15	c	v
Coleoptera	Polyphaga	Staphylinidae	*Phloeocharis subtilissima*	-0.549	-0.149	1.75	c	do
Coleoptera	Polyphaga	Chrysomelidae	*Longitarsus kutscherae*	-0.547	1.361	2	h	v
Coleoptera	Polyphaga	Pselaphidae	*Euplectus bescidicus*	-0.546	-0.143	1.7	c	do
Coleoptera	Polyphaga	Staphylinidae	*Lesteva longoelytrata*	-0.545	-0.735	4	c	g
Coleoptera	Polyphaga	Staphylinidae	*Oxypoda opaca*	-0.545	-0.735	4	c	g
Coleoptera	Polyphaga	Staphylinidae	*Eusphalerum sorbi*	-0.539	1.379	1.85	h	v
Coleoptera	Polyphaga	Pselaphidae	*Plectophloeus fischeri*	-0.538	-0.126	1.55	c	do
Coleoptera	Polyphaga	Cantharidae	*Malthodes pumilus*	-0.530	-0.108	1.4	c	do
Coleoptera	Polyphaga	Chrysomelidae	*Phyllotreta vittula*	-0.528	1.403	1.65	h	v
Coleoptera	Polyphaga	Pselaphidae	*Bibloporus bicolor*	-0.524	-0.096	1.3	c	do
Coleoptera	Polyphaga	Pselaphidae	*Euplectus karsteni*	-0.521	-0.090	1.25	c	do
Coleoptera	Polyphaga	Ptiliidae	*Acrotrichis intermedia*	-0.520	-0.557	1.05	mm	g
Coleoptera	Polyphaga	Pselaphidae	*Bibloporus minutus*	-0.520	-0.087	1.225	c	do
Coleoptera	Polyphaga	Staphylinidae	*Scopaeus laevigatus*	-0.517	-0.676	3.5	c	g
Coleoptera	Polyphaga	Pselaphidae	*Euplectus punctatus*	-0.508	-0.060	1	c	do
Coleoptera	Polyphaga	Anthribidae	*Anthribus albinus*	-0.504	-1.432	9	dx	do
Coleoptera	Polyphaga	Staphylinidae	*Habrocerus capillaricornis*	-0.504	-0.646	3.25	c	g
Coleoptera	Polyphaga	Curculionidae	*Rhinomias forticornis*	-0.481	0.766	2.75	h	g
Coleoptera	Polyphaga	Nitidulidae	*Cryptarcha undata*	-0.480	0.034	2.6	c	ds
Coleoptera	Polyphaga	Pyrochroidae	*Schizotus pectinicornis*	-0.477	-1.372	8.5	dx	do
Coleoptera	Polyphaga	Staphylinidae	*Atheta fungi*	-0.469	-0.569	2.6	c	g
Coleoptera	Polyphaga	Staphylinidae	*Holobus flavicornis*	-0.467	-0.705	1.12	c	e
Coleoptera	Polyphaga	Staphylinidae	*Geostiba circellaris*	-0.466	-0.563	2.55	c	g
Coleoptera	Polyphaga	Staphylinidae	*Syntomium aeneum*	-0.463	-0.557	2.5	c	g
Coleoptera	Polyphaga	Staphylinidae	*Atheta palustris*	-0.460	-0.551	2.45	c	g
Coleoptera	Polyphaga	Elateridae	*Sericus brunneus*	-0.457	-1.185	8.5	dx	v
Coleoptera	Polyphaga	Staphylinidae	*Amischa analis*	-0.441	-0.510	2.1	c	g
Coleoptera	Polyphaga	Pselaphidae	*Brachygluta sinuata*	-0.425	-0.474	1.8	c	g
Coleoptera	Polyphaga	Elateridae	*Ampedus nigrinus*	-0.422	-1.254	7.5	dx	do
Coleoptera	Polyphaga	Cerambycidae	*Stenurella melanura*	-0.422	-1.254	7.5	dx	do
Coleoptera	Polyphaga	Elateridae	*Calambus bipustulatus*	-0.417	-1.242	7.4	dx	do
Coleoptera	Polyphaga	Pselaphidae	*Bryaxis curtisii*	-0.411	-0.445	1.55	c	g

Coleoptera	Polyphaga	Scydmaenidae	*Neuraphes elongatulus*	-0.406	-0.433	1.45	c	g
Coleoptera	Polyphaga	Pselaphidae	*Bryaxis nodicornis*	-0.405	-0.430	1.425	c	g
Coleoptera	Polyphaga	Pselaphidae	*Bryaxis puncticollis*	-0.398	-0.415	1.3	c	g
Coleoptera	Polyphaga	Pselaphidae	*Bythinus macropalpus*	-0.395	-0.409	1.25	c	g
Coleoptera	Polyphaga	Mordellidae	*Tomoxia bucephala*	-0.395	-1.194	7	dx	do
Coleoptera	Polyphaga	Pselaphidae	*Trimium brevicorne*	-0.395	-0.409	1.25	c	g
Coleoptera	Polyphaga	Pselaphidae	*Bythinus burrelli*	-0.392	-0.403	1.2	c	g
Coleoptera	Polyphaga	Elateridae	*Ampedus erythrogonus*	-0.368	-1.135	6.5	dx	do
Coleoptera	Polyphaga	Anobiidae	*Anobium pertinax*	-0.300	-0.987	5.25	dx	do
Coleoptera	Polyphaga	Mordellidae	*Mordellochroa abdominalis*	-0.300	-0.987	5.25	dx	do
Coleoptera	Polyphaga	Curculionidae	*Cossonus cylindricus*	-0.286	-0.957	5	dx	do
Coleoptera	Polyphaga	Eucnemidae	*Eucnemis capucina*	-0.286	-0.957	5	dx	do
Coleoptera	Polyphaga	Alleculidae	*Mycetochara humeralis*	-0.259	-0.898	4.5	dx	do
Coleoptera	Polyphaga	Anobiidae	*Ptilinus pectinicornis*	-0.259	-0.898	4.5	dx	do
Coleoptera	Polyphaga	Scirtidae	*Cyphon coarctatus*	-0.251	1.192	3.75	d	v
Coleoptera	Polyphaga	Anobiidae	*Xestobium plumbeum*	-0.251	-0.880	4.35	dx	do
Coleoptera	Polyphaga	Scarabaeidae	*Aphodius constans*	-0.240	0.478	5.5	d	g
Coleoptera	Polyphaga	Anobiidae	*Hedobia imperialis*	-0.240	-0.856	4.15	dx	do
Coleoptera	Polyphaga	Anobiidae	*Anobium emarginatum*	-0.232	-0.839	4	dx	do
Coleoptera	Polyphaga	Dermestidae	*Megatoma undata*	-0.225	1.141	5	d	ds
Coleoptera	Polyphaga	Curculionidae	*Rhyncolus ater*	-0.218	-0.809	3.75	dx	do
Coleoptera	Polyphaga	Staphylinidae	*Tachinus elongatus*	-0.209	-0.825	8	c	rnf
Coleoptera	Polyphaga	Scraptiidae	*Anaspis frontalis*	-0.199	-0.767	3.4	dx	do
Coleoptera	Polyphaga	Anobiidae	*Ernobius abietis*	-0.180	-0.726	3.05	dx	do
Coleoptera	Polyphaga	Scraptiidae	*Anaspis rufilabris*	-0.177	-0.720	3	dx	do
Coleoptera	Polyphaga	Anobiidae	*Anobium rufipenne*	-0.177	-0.720	3	dx	do
Coleoptera	Polyphaga	Anobiidae	*Episernus granulatus*	-0.177	-0.720	3	dx	do
Coleoptera	Polyphaga	Scraptiidae	*Anaspis ruficollis*	-0.164	-0.690	2.75	dx	do
Coleoptera	Polyphaga	Scraptiidae	*Anaspis thoracica*	-0.164	-0.690	2.75	dx	do
Coleoptera	Polyphaga	Anobiidae	*Ernobius abietinus*	-0.150	-0.661	2.5	dx	do
Coleoptera	Polyphaga	Silphidae	*Oiceoptoma thoracica*	-0.122	-0.087	13.5	d	rnf
Coleoptera	Polyphaga	Staphylinidae	*Philonthus concinnus*	-0.114	-0.618	6.25	c	rnf
Coleoptera	Polyphaga	Nitidulidae	*Glischrochilus quadrisignatus*	-0.100	-0.588	6	c	rnf
Coleoptera	Polyphaga	Histeridae	*Margarinotus striola*	-0.100	-0.588	6	c	rnf
Coleoptera	Polyphaga	Staphylinidae	*Rugilus rufipes*	-0.086	-0.558	5.75	c	rnf
Coleoptera	Polyphaga	Staphylinidae	*Tachinus signatus*	-0.086	-0.558	5.75	c	rnf
Coleoptera	Polyphaga	Ptinidae	*Ptinus subpilosus*	-0.072	0.846	2.4	d	g
Coleoptera	Polyphaga	Dermestidae	*Globicornis nigripes*	-0.062	1.497	2	d	ds
Coleoptera	Polyphaga	Cryptophagidae	*Cryptophagus pubescens*	-0.034	-0.321	2.3	mm	rnf
Coleoptera	Polyphaga	Cryptophagidae	*Cryptophagus thomsoni*	-0.029	-0.309	2.2	mm	rnf
Coleoptera	Polyphaga	Staphylinidae	*Enalodroma hepatica*	-0.018	-0.410	4.5	c	rnf
Coleoptera	Polyphaga	Latridiidae	*Latridius anthracinus*	0.004	-0.238	1.6	mm	rnf
Coleoptera	Polyphaga	Rhizophagidae	*Monotoma longicollis*	0.007	-0.232	1.55	mm	rnf

Coleoptera	Polyphaga	Clambidae	*Clambus armadillo*	0.029	-0.184	1.15	mm	rnf
Coleoptera	Polyphaga	Staphylinidae	*Oxypoda alternans*	0.036	-0.291	3.5	c	rnf
Coleoptera	Polyphaga	Ptiliidae	*Acrotrichis grandicollis*	0.039	-0.160	0.95	mm	rnf
Coleoptera	Polyphaga	Ptiliidae	*Acrotrichis insularis*	0.042	-0.155	0.9	mm	rnf
Coleoptera	Polyphaga	Staphylinidae	*Aleochara bipustulata*	0.050	-0.262	3.25	c	rnf
Coleoptera	Polyphaga	Staphylinidae	*Atheta crassicornis*	0.058	-0.244	3.1	c	rnf
Coleoptera	Adephaga	Dytiscidae	*Hydroporus palustris*	0.058	-0.244	3.1	c	rnf
Coleoptera	Polyphaga	Staphylinidae	*Haploglossa villosula*	0.063	-0.232	3	c	rnf
Coleoptera	Polyphaga	Histeridae	*Gnathoncus buyssoni*	0.074	-0.208	2.8	c	rnf
Coleoptera	Polyphaga	Staphylinidae	*Atheta sodalis*	0.077	-0.202	2.75	c	rnf
Coleoptera	Polyphaga	Staphylinidae	*Atheta nigricornis*	0.082	-0.191	2.65	c	rnf
Coleoptera	Polyphaga	Staphylinidae	*Atheta subtilis*	0.107	-0.137	2.2	c	rnf
Coleoptera	Polyphaga	Staphylinidae	*Atheta cauta*	0.112	-0.125	2.1	c	rnf
Coleoptera	Polyphaga	Staphylinidae	*Atheta aegra*	0.131	-0.084	1.75	c	rnf
Coleoptera	Polyphaga	Corylophidae	*Sericoderus lateralis*	0.161	-0.019	1.2	c	rnf
Coleoptera	Polyphaga	Tenebrionidae	*Bolitophagus reticulatus*	0.182	-1.129	6.5	m	wf
Coleoptera	Polyphaga	Geotrupidae	*Anoplotrupes stercorosus*	0.204	0.625	7.5	d	rnf
Coleoptera	Polyphaga	Nitidulidae	*Cychramus variegatus*	0.209	-1.069	6	m	wf
Coleoptera	Polyphaga	Mycetophagidae	*Mycetophagus quadripustulatus*	0.236	-1.010	5.5	m	wf
Coleoptera	Polyphaga	Trogidae	*Trox scaber*	0.259	0.744	6.5	d	rnf
Coleoptera	Polyphaga	Staphylinidae	*Coprophilus striatulus*	0.286	0.803	6	d	rnf
Coleoptera	Polyphaga	Cholevidae	*Catops picipes*	0.300	0.833	5.75	d	rnf
Coleoptera	Polyphaga	Nitidulidae	*Cychramus luteus*	0.302	-0.868	4.3	m	wf
Coleoptera	Polyphaga	Mycetophagidae	*Mycetophagus atomarius*	0.304	-0.862	4.25	m	wf
Coleoptera	Polyphaga	Mycetophagidae	*Mycetophagus piceus*	0.318	-0.832	4	m	wf
Coleoptera	Polyphaga	Mycetophagidae	*Mycetophagus quadriguttatus*	0.332	-0.802	3.75	m	wf
Coleoptera	Polyphaga	Erotylidae	*Tritoma bipustulata*	0.332	-0.802	3.75	m	wf
Coleoptera	Polyphaga	Cisidae	*Cis boleti*	0.372	-0.713	3	m	wf
Coleoptera	Polyphaga	Cleridae	*Thanasimus formicarius*	0.380	-1.357	8.5	c	df
Coleoptera	Polyphaga	Mycetophagidae	*Litargus connexus*	0.394	-0.666	2.6	m	wf
Coleoptera	Polyphaga	Leiodidae	*Anisotoma orbicularis*	0.400	-0.654	2.5	m	wf
Coleoptera	Polyphaga	Cisidae	*Cis punctulatus*	0.400	-0.654	2.5	m	wf
Coleoptera	Polyphaga	Latridiidae	*Stephostethus alternans*	0.400	-0.654	2.5	m	wf
Coleoptera	Polyphaga	Cisidae	*Orthocis alni*	0.408	-0.636	2.35	m	wf
Coleoptera	Polyphaga	Cisidae	*Cis dentatus*	0.411	-0.630	2.3	m	wf
Coleoptera	Polyphaga	Cisidae	*Cis hispidus*	0.416	-0.619	2.2	m	wf
Coleoptera	Polyphaga	Cisidae	*Orthocis festivus*	0.416	-0.619	2.2	m	wf
Coleoptera	Polyphaga	Staphylinidae	*Scaphisoma agaricinum*	0.416	-0.619	2.2	m	wf
Coleoptera	Polyphaga	Cisidae	*Cis castaneus*	0.432	-0.583	1.9	m	wf
Coleoptera	Polyphaga	Latridiidae	*Latridius hirtus*	0.432	-0.583	1.9	m	wf
Coleoptera	Polyphaga	Cisidae	*Orthocis vestitus*	0.435	-0.577	1.85	m	wf
Coleoptera	Polyphaga	Cerambycidae	*Rhagium inquisitor*	0.439	-2.610	15.5	dx	df
Coleoptera	Polyphaga	Cisidae	*Ennearthron cornutum*	0.443	-0.559	1.7	m	wf

Coleoptera	Polyphaga	Staphylinidae	*Omalium caesum*	0.449	1.159	3	d	rnf
Coleoptera	Polyphaga	Staphylinidae	*Omalium rivulare*	0.449	1.159	3	d	rnf
Coleoptera	Polyphaga	Cholevidae	*Sciodrepoides watsoni*	0.449	1.159	3	d	rnf
Coleoptera	Polyphaga	Colydiidae	*Synchita humeralis*	0.465	0.588	3	m	do
Coleoptera	Polyphaga	Malachidae	*Anthocomus fasciatus*	0.472	0.746	3.25	m	v
Coleoptera	Polyphaga	Cleridae	*Thanasimus pectoralis*	0.475	-1.150	6.75	c	df
Coleoptera	Polyphaga	Cisidae	*Orthocis pygmaeus*	0.481	-0.476	1	m	wf
Coleoptera	Polyphaga	Staphylinidae	*Anotylus tetracarinatus*	0.506	1.284	1.95	d	rnf
Coleoptera	Polyphaga	Hydrophilidae	*Megasternum obscurum*	0.506	1.284	1.95	d	rnf
Coleoptera	Polyphaga	Throscidae	*Trixagus dermestoides*	0.510	-0.048	3.05	m	e
Coleoptera	Polyphaga	Cerambycidae	*Tetropium castaneum*	0.547	-2.373	13.5	dx	df
Coleoptera	Polyphaga	Throscidae	*Trixagus leseigneuri*	0.567	0.076	2.0	m	NA
Coleoptera	Polyphaga	Throscidae	*Trixagus meybohmi*	0.567	0.076	2.0	m	NA
Coleoptera	Polyphaga	Trogositidae	*Nemosoma elongatum*	0.571	-0.942	5	c	df
Coleoptera	Polyphaga	Nitidulidae	*Pityophagus ferrugineus*	0.571	-0.942	5	c	df
Coleoptera	Polyphaga	Nitidulidae	*Glischrochilus quadripunctatus*	0.584	-0.913	4.75	c	df
Coleoptera	Polyphaga	Throscidae	*Aulonothroscus brevicollis*	0.605	0.299	2.75	m	g
Coleoptera	Polyphaga	Cerambycidae	*Callidium aeneum*	0.629	-2.195	12	dx	df
Coleoptera	Polyphaga	Lymexylonidae	*Hylecoetus dermestoides*	0.629	-2.195	12	dx	df
Coleoptera	Polyphaga	Salpingidae	*Salpingus ruficollis*	0.630	-0.812	3.9	c	df
Coleoptera	Polyphaga	Endomychidae	*Symbiotes latus*	0.634	0.991	2	m	ds
Coleoptera	Polyphaga	Rhizophagidae	*Rhizophagus ferrugineus*	0.639	-0.794	3.75	c	df
Coleoptera	Polyphaga	Rhizophagidae	*Rhizophagus dispar*	0.652	-0.764	3.5	c	df
Coleoptera	Polyphaga	Rhizophagidae	*Rhizophagus depressus*	0.658	-0.752	3.4	c	df
Coleoptera	Polyphaga	Salpingidae	*Salpingus planirostris*	0.666	-0.735	3.25	c	df
Coleoptera	Polyphaga	Nitidulidae	*Epuraea marseuli*	0.679	-0.705	3	c	df
Coleoptera	Polyphaga	Laemophloeidae	*Leptophloeus alternans*	0.679	-0.705	3	c	df
Coleoptera	Polyphaga	Rhizophagidae	*Rhizophagus perforatus*	0.679	-0.705	3	c	df
Coleoptera	Polyphaga	Salpingidae	*Vincenzellus ruficollis*	0.679	-0.705	3	c	df
Coleoptera	Polyphaga	Cerambycidae	*Clytus lama*	0.683	-2.076	11	dx	df
Coleoptera	Polyphaga	Cerambycidae	*Molorchus minor*	0.683	-2.076	11	dx	df
Coleoptera	Polyphaga	Rhizophagidae	*Rhizophagus bipustulatus*	0.685	-0.693	2.9	c	df
Coleoptera	Polyphaga	Endomychidae	*Symbiotes gibberosus*	0.688	1.110	1	m	ds
Coleoptera	Polyphaga	Staphylinidae	*Phloeopora testacea*	0.693	-0.675	2.75	c	df
Coleoptera	Polyphaga	Staphylinidae	*Phloeopora corticalis*	0.704	-0.652	2.55	c	df
Coleoptera	Polyphaga	Staphylinidae	*Ischnoglossa spec.*	0.707	-0.646	2.5	c	df
Coleoptera	Polyphaga	Cerambycidae	*Clytus arietis*	0.711	-2.017	10.5	dx	df
Coleoptera	Polyphaga	Nitidulidae	*Epuraea pygmaea*	0.720	-0.616	2.25	c	df
Coleoptera	Polyphaga	Curculionidae	*Hylobius abietis*	0.724	-1.987	10.25	dx	df
Coleoptera	Polyphaga	Staphylinidae	*Placusa tachyporoides*	0.734	-0.586	2	c	df
Coleoptera	Polyphaga	Cerambycidae	*Anaglyptus mysticus*	0.765	-1.898	9.5	dx	df
Coleoptera	Polyphaga	Curculionidae	*Pissodes pini*	0.882	-1.643	7.35	dx	df
Coleoptera	Polyphaga	Cerambycidae	*Obrium brunneum*	0.983	-1.424	5.5	dx	df

Coleoptera	Polyphaga	Cerambycidae	*Pogonocherus decoratus*	1.010	-1.364	5	dx	df
Coleoptera	Polyphaga	Curculionidae	*Magdalis phlegmatica*	1.015	-1.353	4.9	dx	df
Coleoptera	Polyphaga	Scolytidae	*Ips typographus*	1.018	-1.347	4.85	dx	df
Coleoptera	Polyphaga	Scolytidae	*Dryocoetes autographus*	1.072	-1.228	3.85	dx	df
Coleoptera	Polyphaga	Scolytidae	*Hylastes cunicularius*	1.072	-1.228	3.85	dx	df
Coleoptera	Polyphaga	Scolytidae	*Dryocoetes villosus*	1.119	-1.127	3	dx	df
Coleoptera	Polyphaga	Scolytidae	*Leperisinus fraxini*	1.119	-1.127	3	dx	df
Coleoptera	Polyphaga	Scolytidae	*Scolytus intricatus*	1.119	-1.127	3	dx	df
Coleoptera	Polyphaga	Scolytidae	*Hylurgops palliatus*	1.127	-1.109	2.85	dx	df
Coleoptera	Polyphaga	Scolytidae	*Polygraphus poligraphus*	1.140	-1.080	2.6	dx	df
Coleoptera	Polyphaga	Leiodidae	*Agathidium varians*	1.159	0.683	2.75	m	rnf
Coleoptera	Polyphaga	Scolytidae	*Pityogenes chalcographus*	1.165	-1.026	2.15	dx	df
Coleoptera	Polyphaga	Anobiidae	*Dryophilus pusillus*	1.168	-1.020	2.1	dx	df
Coleoptera	Polyphaga	Scolytidae	*Taphrorychus bicolor*	1.170	-1.015	2.05	dx	df
Coleoptera	Polyphaga	Leiodidae	*Agathidium seminulum*	1.186	0.743	2.25	m	rnf
Coleoptera	Polyphaga	Scolytidae	*Ernoporicus fagi*	1.187	-0.979	1.75	dx	df
Coleoptera	Polyphaga	Scolytidae	*Cryphalus piceae*	1.203	-0.943	1.45	dx	df
Coleoptera	Polyphaga	Scolytidae	*Pityophthorus pityographus*	1.208	-0.931	1.35	dx	df
Coleoptera	Polyphaga	Scolytidae	*Crypturgus cinereus*	1.211	-0.926	1.3	dx	df
Coleoptera	Polyphaga	Scolytidae	*Pityophthorus exsculptus*	1.211	-0.926	1.3	dx	df
Coleoptera	Polyphaga	Scolytidae	*Crypturgus hispidulus*	1.214	-0.920	1.25	dx	df
Coleoptera	Polyphaga	Scolytidae	*Xyloterus domesticus*	1.737	0.127	3.45	m	df
Coleoptera	Polyphaga	Scolytidae	*Xyloterus lineatus*	1.750	0.157	3.2	m	df
Coleoptera	Polyphaga	Scolytidae	*Gnathotrichus materiarius*	1.761	0.181	3	m	df
Coleoptera	Polyphaga	Scolytidae	*Xyleborus monographus*	1.815	0.299	2	m	df
Coleoptera	Polyphaga	Scolytidae	*Xyleborus peregrinus*	1.815	0.299	2	m	df
Coleoptera	Polyphaga	Scolytidae	*Xyleborus dispar*	1.818	0.305	1.95	m	df
Coleoptera	Polyphaga	Scolytidae	*Xyleborus saxeseni*	1.826	0.323	1.8	m	df
Coleoptera	Polyphaga	Scolytidae	*Xyleborus germanus*	1.848	0.371	1.4	m	df

Feeding guilds: c = carnivore, d = decomposer (excl. wood), dx = decomposer-wood, h = herbivore (excl. xylophage), m = mycetophagous-fungi, mm = mycetophagous-mold

Habitat guilds: df = fresh dead wood, do = old dead wood, ds = specific dead wood structures at living trees, e = eurytop, g = ground dweller, rnf = rotten substrate/nests/fungi (excl. wood), v = vegetation, wf = wood fungi.

Table A: Multivariate fourth corner analyses [1] on the effects of Sampling solution, stratum, and forest type on species traits. We used the sequential test [2] as recommended in Dray et al. [3], using Holm correction of p-values (999 permutations). Significant value is marked in bold.

	Statistic	Sampling Solution	Stratum	Forest type
**Mean body size**	F	30.316	45.452	144.514
**Feeding guild**	Chi²	646.494	216.936	233.906
**Habitat preference**	Chi²	**1151.755****	267.656	116.681

References

1. Dray S, Legendre P (2008) Testing the species traits-environment relationships: the fourth-corner problem revisited. Ecology 89: 3400-3412.
2. ter Braak CJF, Cormont A, Dray S (2012) Improved testing of species traits–environment relationships in the fourth-corner problem. Ecology 93: 1525-1526.
3. Dray S, Choler P, Dolédec S, Peres-Neto PR, Thuiller W, Pavoine S, et al. (2013) Combining the fourth-corner and the RLQ methods for assessing trait responses to environmental variation. Ecology 95: 14-21.
