# Supplementary material for: Searching for the Optimal Sampling Solution: Variation in Invertebrate Communities, Sample Condition and DNA Quality
Source: PLoS One. 2016 Feb 3;11(2):e0148247. doi: 10.1371/journal.pone.0148247 (PMC4740435; doi:10.1371/journal.pone.0148247)
Supplement: S7 File — Table A gives a list of species including abundance sampled over seven weeks between May and July 2013, separated by forest type, stratum and top and bottom jars of flight-interception traps. (PDF) [file pone.0148247.s007.pdf]

Supplementary S7: Overview of sampled species

Table A: List of species including abundance sampled over seven weeks between May and July 2013, separated by forest type, stratum and top and bottom jars of flight-interception traps.

				Beech forest				Spruce forest				
				Understorey		Canopy		Understorey		Canopy		
**Order**	**Suborder**	**Family**	**SpeciesID**	**Bottom**	**Top**	**Bottom**	**Top**	**Bottom**	**Top**	**Bottom**	**Top**	**Total**
Coleoptera	Adephaga	Carabidae	*Amara ovata*	0	0	0	0	1	0	0	0	1
Coleoptera	Adephaga	Carabidae	*Amara plebeja*	0	0	0	0	1	0	0	0	1
Coleoptera	Adephaga	Carabidae	*Amara similata*	0	0	0	0	1	0	0	0	1
Coleoptera	Adephaga	Carabidae	*Dromius agilis*	0	0	3	0	0	0	8	0	11
Coleoptera	Adephaga	Carabidae	*Pterostichus vernalis*	0	0	0	0	1	0	0	0	1
Coleoptera	Adephaga	Dytiscidae	*Hydroporus palustris*	0	0	0	0	1	0	0	0	1
Coleoptera	Polyphaga	Alleculidae	*Mycetochara humeralis*	0	0	2	0	0	0	0	0	2
Coleoptera	Polyphaga	Anobiidae	*Anobium emarginatum*	0	0	0	0	0	0	3	0	3
Coleoptera	Polyphaga	Anobiidae	*Anobium pertinax*	0	0	0	0	1	0	0	0	1
Coleoptera	Polyphaga	Anobiidae	*Anobium rufipenne*	4	0	21	1	0	0	0	0	26
Coleoptera	Polyphaga	Anobiidae	*Dryophilus pusillus*	0	0	0	0	3	0	7	0	10
Coleoptera	Polyphaga	Anobiidae	*Episernus granulatus*	0	0	0	0	0	0	1	0	1
Coleoptera	Polyphaga	Anobiidae	*Ernobius abietinus*	0	0	0	0	5	0	6	1	12
Coleoptera	Polyphaga	Anobiidae	*Ernobius abietis*	1	0	0	0	0	0	1	0	2
Coleoptera	Polyphaga	Anobiidae	*Hedobia imperialis*	5	0	4	0	0	0	1	0	10
Coleoptera	Polyphaga	Anobiidae	*Ptilinus pectinicornis*	3	0	4	0	0	0	1	0	8
Coleoptera	Polyphaga	Anobiidae	*Xestobium plumbeum*	0	0	2	1	0	0	0	0	3
Coleoptera	Polyphaga	Anthribidae	*Anthribus albinus*	4	0	4	1	5	0	4	1	19
Coleoptera	Polyphaga	Anthribidae	*Brachytarsus nebulosus*	0	0	0	0	3	2	4	0	9
Coleoptera	Polyphaga	Bothrideridae	*Oxylaemus variolosus*	2	0	0	0	0	0	0	0	2
Coleoptera	Polyphaga	Byturidae	*Byturus ochraceus*	4	0	0	0	7	0	0	0	11

Coleoptera	Polyphaga	Byturidae	*Byturus tomentosus*	5	0	12	0	43	1	20	0	81
Coleoptera	Polyphaga	Cantharidae	*Cantharis decipiens*	2	0	0	0	0	0	0	0	2
Coleoptera	Polyphaga	Cantharidae	*Cantharis nigricans*	0	1	1	1	3	2	3	1	12
Coleoptera	Polyphaga	Cantharidae	*Cantharis pellucida*	1	2	2	14	4	5	19	8	55
Coleoptera	Polyphaga	Cantharidae	*Malthinus frontalis*	0	0	2	0	2	0	1	0	5
Coleoptera	Polyphaga	Cantharidae	*Malthodes marginatus*	0	0	1	0	0	0	0	0	1
Coleoptera	Polyphaga	Cantharidae	*Malthodes pumilus*	2	1	3	1	6	2	2	0	17
Coleoptera	Polyphaga	Cantharidae	*Malthodes spathifer*	2	0	0	1	4	0	2	0	9
Coleoptera	Polyphaga	Cantharidae	*Metacantharis discoidea*	0	0	0	0	1	20	4	4	29
Coleoptera	Polyphaga	Cantharidae	*Podabrus alpinus*	0	0	0	0	1	0	0	1	2
Coleoptera	Polyphaga	Cantharidae	*Rhagonycha atra*	0	0	0	0	2	3	1	0	6
Coleoptera	Polyphaga	Cantharidae	*Rhagonycha lignosa*	3	0	1	0	6	0	3	0	13
Coleoptera	Polyphaga	Cantharidae	*Rhagonycha testacea*	0	0	0	0	3	0	0	0	3
Coleoptera	Polyphaga	Cantharidae	*Rhagonycha translucida*	0	0	0	1	0	0	0	0	1
Coleoptera	Polyphaga	Cerambycidae	*Anaglyptus mysticus*	0	1	0	1	0	0	0	0	2
Coleoptera	Polyphaga	Cerambycidae	*Callidium aeneum*	0	0	0	0	0	0	2	1	3
Coleoptera	Polyphaga	Cerambycidae	*Clytus arietis*	0	1	0	0	0	0	0	0	1
Coleoptera	Polyphaga	Cerambycidae	*Clytus lama*	0	0	0	0	0	0	1	0	1
Coleoptera	Polyphaga	Cerambycidae	*Molorchus minor*	0	0	0	0	3	0	7	0	10
Coleoptera	Polyphaga	Cerambycidae	*Obrium brunneum*	0	0	0	0	0	0	2	0	2
Coleoptera	Polyphaga	Cerambycidae	*Oxymirus cursor*	0	0	0	0	3	1	0	0	4
Coleoptera	Polyphaga	Cerambycidae	*Pogonocherus decoratus*	0	0	0	0	0	0	1	0	1
Coleoptera	Polyphaga	Cerambycidae	*Rhagium inquisitor*	0	0	0	0	16	0	2	0	18
Coleoptera	Polyphaga	Cerambycidae	*Stenurella melanura*	0	0	0	0	1	0	0	0	1
Coleoptera	Polyphaga	Cerambycidae	*Tetropium castaneum*	0	0	0	0	7	0	0	0	7
Coleoptera	Polyphaga	Cerylonidae	*Cerylon ferrugineum*	1	0	0	0	1	0	1	0	3
Coleoptera	Polyphaga	Cerylonidae	*Cerylon histeroides*	2	1	0	0	2	0	0	0	5
Coleoptera	Polyphaga	Cholevidae	*Catops picipes*	1	0	0	0	0	0	0	0	1
Coleoptera	Polyphaga	Cholevidae	*Sciodrepoides watsoni*	7	0	0	0	1	0	0	0	8
Coleoptera	Polyphaga	Chrysomelidae	*Longitarsus kutscherae*	0	0	0	0	0	1	0	0	1

Coleoptera	Polyphaga	Chrysomelidae	*Oulema melanopus*	0	0	0	0	1	0	0	0	1
Coleoptera	Polyphaga	Chrysomelidae	*Phyllotreta vittula*	0	0	0	0	0	1	0	0	1
Coleoptera	Polyphaga	Cimberidae	*Cimberis attelaboides*	0	0	0	0	2	0	9	0	11
Coleoptera	Polyphaga	Cisidae	*Cis boleti*	1	0	0	0	0	0	0	0	1
Coleoptera	Polyphaga	Cisidae	*Cis castaneus*	4	1	0	0	0	0	0	0	5
Coleoptera	Polyphaga	Cisidae	*Cis dentatus*	0	0	0	0	1	0	0	0	1
Coleoptera	Polyphaga	Cisidae	*Cis hispidus*	0	0	2	0	1	0	0	0	3
Coleoptera	Polyphaga	Cisidae	*Cis punctulatus*	0	0	0	0	4	0	0	0	4
Coleoptera	Polyphaga	Cisidae	*Ennearthron cornutum*	1	0	0	2	0	0	1	0	4
Coleoptera	Polyphaga	Cisidae	*Orthocis alni*	1	0	0	0	0	0	0	0	1
Coleoptera	Polyphaga	Cisidae	*Orthocis festivus*	1	0	0	0	0	0	0	0	1
Coleoptera	Polyphaga	Cisidae	*Orthocis pygmaeus*	1	0	0	0	2	0	0	0	3
Coleoptera	Polyphaga	Cisidae	*Orthocis vestitus*	2	2	0	0	2	0	0	0	6
Coleoptera	Polyphaga	Clambidae	*Clambus armadillo*	0	0	0	0	0	0	1	0	1
Coleoptera	Polyphaga	Cleridae	*Opilo mollis*	0	1	3	2	0	0	1	2	9
Coleoptera	Polyphaga	Cleridae	*Thanasimus formicarius*	1	0	0	0	5	6	1	0	13
Coleoptera	Polyphaga	Cleridae	*Thanasimus pectoralis*	0	0	0	0	0	0	1	0	1
Coleoptera	Polyphaga	Coccinellidae	*Adalia decempunctata*	0	0	0	0	0	1	1	0	2
Coleoptera	Polyphaga	Coccinellidae	*Anatis ocellata*	0	0	0	0	0	0	0	1	1
Coleoptera	Polyphaga	Coccinellidae	*Aphidecta obliterata*	0	0	0	0	0	0	1	0	1
Coleoptera	Polyphaga	Coccinellidae	*Exochomus quadripustulatus*	0	0	0	0	1	0	0	0	1
Coleoptera	Polyphaga	Coccinellidae	*Harmonia axyridis*	0	1	0	0	0	2	0	0	3
Coleoptera	Polyphaga	Coccinellidae	*Myzia oblongoguttata*	0	0	0	0	1	0	0	1	2
Coleoptera	Polyphaga	Coccinellidae	*Rhyzobius chrysomeloides*	0	0	0	0	1	1	0	0	2
Coleoptera	Polyphaga	Coccinellidae	*Scymnus abietis*	0	0	0	0	1	0	1	0	2
Coleoptera	Polyphaga	Colydiidae	*Synchita humeralis*	0	0	0	0	1	0	0	0	1
Coleoptera	Polyphaga	Corylophidae	*Orthoperus atomus*	0	0	0	0	10	1	21	0	32
Coleoptera	Polyphaga	Corylophidae	*Orthoperus mundus*	0	0	0	0	1	0	0	0	1
Coleoptera	Polyphaga	Corylophidae	*Sericoderus lateralis*	1	0	0	0	2	0	0	0	3
Coleoptera	Polyphaga	Cryptophagidae	*Atomaria analis*	0	0	0	0	1	0	0	0	1

Coleoptera	Polyphaga	Cryptophagidae	*Atomaria atricapilla*	2	2	0	0	0	0	0	0	4
Coleoptera	Polyphaga	Cryptophagidae	*Atomaria fuscata*	2	1	0	0	0	0	0	0	3
Coleoptera	Polyphaga	Cryptophagidae	*Atomaria nigrirostris*	0	0	0	0	1	0	0	0	1
Coleoptera	Polyphaga	Cryptophagidae	*Atomaria ornata*	0	0	0	0	7	0	0	0	7
Coleoptera	Polyphaga	Cryptophagidae	*Atomaria procerula*	0	0	0	0	1	0	0	0	1
Coleoptera	Polyphaga	Cryptophagidae	*Atomaria turgida*	0	0	0	0	32	0	0	0	32
Coleoptera	Polyphaga	Cryptophagidae	*Cryptophagus dentatus*	2	0	0	0	0	0	2	0	4
Coleoptera	Polyphaga	Cryptophagidae	*Cryptophagus distinguendus*	1	0	0	0	1	0	0	0	2
Coleoptera	Polyphaga	Cryptophagidae	*Cryptophagus dorsalis*	0	0	0	0	0	0	2	0	2
Coleoptera	Polyphaga	Cryptophagidae	*Cryptophagus pilosus*	1	0	0	0	2	0	0	0	3
Coleoptera	Polyphaga	Cryptophagidae	*Cryptophagus pubescens*	2	0	2	0	1	0	0	0	5
Coleoptera	Polyphaga	Cryptophagidae	*Cryptophagus scanicus*	1	0	0	0	0	0	0	0	1
Coleoptera	Polyphaga	Cryptophagidae	*Cryptophagus thomsoni*	1	0	0	0	0	0	0	0	1
Coleoptera	Polyphaga	Cryptophagidae	*Micrambe abietis*	0	0	0	0	16	1	11	0	28
Coleoptera	Polyphaga	Curculionidae	*Anthonomus phyllocola*	0	0	0	0	0	0	1	0	1
Coleoptera	Polyphaga	Curculionidae	*Barypeithes pellucidus*	28	0	0	0	0	0	0	0	28
Coleoptera	Polyphaga	Curculionidae	*Cossonus cylindricus*	0	0	1	0	0	0	0	0	1
Coleoptera	Polyphaga	Curculionidae	*Curculio glandium*	0	2	1	1	0	0	0	0	4
Coleoptera	Polyphaga	Curculionidae	*Curculio pellitus*	0	1	0	0	0	0	0	0	1
Coleoptera	Polyphaga	Curculionidae	*Curculio venosus*	0	1	0	0	0	0	0	0	1
Coleoptera	Polyphaga	Curculionidae	*Hylobius abietis*	0	0	0	0	0	0	4	0	4
Coleoptera	Polyphaga	Curculionidae	*Hypera nigrirostris*	0	0	0	0	1	0	0	0	1
Coleoptera	Polyphaga	Curculionidae	*Magdalis phlegmatica*	0	0	0	0	1	0	0	0	1
Coleoptera	Polyphaga	Curculionidae	*Otiorhynchus scaber*	0	0	0	0	2	0	1	0	3
Coleoptera	Polyphaga	Curculionidae	*Otiorhynchus singularis*	0	0	0	0	5	0	0	0	5
Coleoptera	Polyphaga	Curculionidae	*Phyllobius arborator*	0	0	0	0	2	1	0	0	3
Coleoptera	Polyphaga	Curculionidae	*Phyllobius argentatus*	6	0	4	0	0	1	0	0	11
Coleoptera	Polyphaga	Curculionidae	*Phyllobius pomaceus*	1	0	0	0	0	0	0	0	1
Coleoptera	Polyphaga	Curculionidae	*Pissodes pini*	0	0	0	0	0	1	0	0	1
Coleoptera	Polyphaga	Curculionidae	*Polydrusus impar*	0	0	0	0	8	15	9	3	35

Coleoptera	Polyphaga	Curculionidae	*Polydrusus undatus*	5	5	6	4	0	0	1	1	22
Coleoptera	Polyphaga	Curculionidae	*Rhinomias forticornis*	0	0	0	0	1	0	0	0	1
Coleoptera	Polyphaga	Curculionidae	*Rhynchaenus fagi*	146	14	175	8	8	4	13	0	368
Coleoptera	Polyphaga	Curculionidae	*Rhynchaenus quercus*	0	0	0	0	1	0	3	0	4
Coleoptera	Polyphaga	Curculionidae	*Rhyncolus ater*	0	0	0	0	1	0	0	0	1
Coleoptera	Polyphaga	Curculionidae	*Stereonychus fraxini*	0	0	1	0	0	0	0	0	1
Coleoptera	Polyphaga	Curculionidae	*Strophosoma melanogrammum*	8	0	3	0	11	0	0	0	22
Coleoptera	Polyphaga	Dermestidae	*Globicornis nigripes*	0	0	1	0	0	0	0	0	1
Coleoptera	Polyphaga	Dermestidae	*Megatoma undata*	0	0	3	0	3	0	4	1	11
Coleoptera	Polyphaga	Elateridae	*Agriotes acuminatus*	6	0	1	0	63	0	5	0	75
Coleoptera	Polyphaga	Elateridae	*Agriotes pallidulus*	0	0	2	0	0	0	0	0	2
Coleoptera	Polyphaga	Elateridae	*Agriotes pilosellus*	7	0	0	0	1	0	0	0	8
Coleoptera	Polyphaga	Elateridae	*Ampedus erythrogonus*	0	0	0	0	2	0	6	0	8
Coleoptera	Polyphaga	Elateridae	*Ampedus nigrinus*	0	0	0	0	2	0	5	0	7
Coleoptera	Polyphaga	Elateridae	*Ampedus pomorum*	1	0	1	0	0	0	1	0	3
Coleoptera	Polyphaga	Elateridae	*Ampedus sanguineus*	0	0	1	0	0	0	0	0	1
Coleoptera	Polyphaga	Elateridae	*Anostirus purpureus*	0	0	0	0	1	0	0	0	1
Coleoptera	Polyphaga	Elateridae	*Athous haemorrhoidalis*	0	0	0	0	1	0	0	0	1
Coleoptera	Polyphaga	Elateridae	*Athous subfuscus*	94	1	45	1	302	1	165	2	611
Coleoptera	Polyphaga	Elateridae	*Athous vittatus*	46	1	43	1	12	0	5	0	108
Coleoptera	Polyphaga	Elateridae	*Calambus bipustulatus*	0	0	3	0	0	0	0	0	3
Coleoptera	Polyphaga	Elateridae	*Dalopius marginatus*	18	0	22	3	153	1	116	0	313
Coleoptera	Polyphaga	Elateridae	*Denticollis linearis*	12	0	1	0	2	0	0	0	15
Coleoptera	Polyphaga	Elateridae	*Kibunea minutus*	0	0	0	0	0	0	1	0	1
Coleoptera	Polyphaga	Elateridae	*Melanotus castanipes*	2	0	0	0	21	0	5	0	28
Coleoptera	Polyphaga	Elateridae	*Melanotus rufipes*	5	0	1	0	8	0	4	0	18
Coleoptera	Polyphaga	Elateridae	*Sericus brunneus*	0	0	0	0	0	0	1	0	1
Coleoptera	Polyphaga	Endomychidae	*Symbiotes gibberosus*	0	0	1	0	0	0	0	0	1
Coleoptera	Polyphaga	Endomychidae	*Symbiotes latus*	0	0	0	1	1	0	0	0	2
Coleoptera	Polyphaga	Erotylidae	*Tritoma bipustulata*	0	0	0	0	2	0	1	0	3

Coleoptera	Polyphaga	Eucnemidae	*Eucnemis capucina*	0	0	1	0	0	0	0	0	1
Coleoptera	Polyphaga	Geotrupidae	*Anoplotrupes stercorosus*	0	0	0	0	2	0	0	0	2
Coleoptera	Polyphaga	Histeridae	*Gnathoncus buyssoni*	0	0	2	0	0	0	0	0	2
Coleoptera	Polyphaga	Histeridae	*Margarinotus striola*	1	0	0	0	1	0	0	0	2
Coleoptera	Polyphaga	Histeridae	*Paromalus flavicornis*	2	0	0	0	0	0	0	0	2
Coleoptera	Polyphaga	Hydrophilidae	*Megasternum obscurum*	0	0	0	0	2	0	0	0	2
Coleoptera	Polyphaga	Laemophloeidae	*Leptophloeus alternans*	0	0	0	0	0	0	2	0	2
Coleoptera	Polyphaga	Latridiidae	*Cartodere constricta*	0	0	0	0	8	0	0	0	8
Coleoptera	Polyphaga	Latridiidae	*Cartodere nodifer*	3	1	0	0	1	0	0	0	5
Coleoptera	Polyphaga	Latridiidae	*Corticaria abietorum*	1	0	0	0	7	0	24	0	32
Coleoptera	Polyphaga	Latridiidae	*Corticarina fuscula*	0	0	0	0	1	0	0	0	1
Coleoptera	Polyphaga	Latridiidae	*Corticarina lambiana*	0	0	0	0	37	3	37	0	77
Coleoptera	Polyphaga	Latridiidae	*Corticarina similata*	1	2	3	1	5	1	24	5	42
Coleoptera	Polyphaga	Latridiidae	*Cortinicara gibbosa*	9	3	13	9	7	2	7	0	50
Coleoptera	Polyphaga	Latridiidae	*Dienerella clathrata*	0	0	0	0	1	0	0	0	1
Coleoptera	Polyphaga	Latridiidae	*Enicmus histrio*	1	0	0	0	0	0	0	0	1
Coleoptera	Polyphaga	Latridiidae	*Enicmus transversus*	0	0	0	0	1	0	0	0	1
Coleoptera	Polyphaga	Latridiidae	*Latridius anthracinus*	0	0	0	0	1	0	0	0	1
Coleoptera	Polyphaga	Latridiidae	*Latridius hirtus*	0	0	1	0	0	0	0	0	1
Coleoptera	Polyphaga	Latridiidae	*Stephostethus alternans*	4	0	0	0	1	0	0	0	5
Coleoptera	Polyphaga	Latridiidae	*Stephostethus angusticollis*	2	0	0	0	2	0	0	0	4
Coleoptera	Polyphaga	Latridiidae	*Stephostethus rugicollis*	0	0	0	0	5	0	0	0	5
Coleoptera	Polyphaga	Leiodidae	*Agathidium seminulum*	3	0	1	0	44	0	0	0	48
Coleoptera	Polyphaga	Leiodidae	*Agathidium varians*	1	0	0	0	0	0	0	0	1
Coleoptera	Polyphaga	Leiodidae	*Anisotoma orbicularis*	0	0	0	0	1	0	0	0	1
Coleoptera	Polyphaga	Lycidae	*Dictyopterus aurora*	0	0	0	0	3	0	0	0	3
Coleoptera	Polyphaga	Lymexylonidae	*Hylecoetus dermestoides*	32	0	0	0	114	0	0	0	146
Coleoptera	Polyphaga	Malachidae	*Anthocomus fasciatus*	0	0	1	0	0	0	0	0	1
Coleoptera	Polyphaga	Melyridae	*Aplocnemus nigricornis*	0	0	0	1	0	0	2	0	3
Coleoptera	Polyphaga	Melyridae	*Dasytes aeratus*	0	0	1	0	0	0	0	0	1

Coleoptera	Polyphaga	Melyridae	*Dasytes caeruleus*	5	0	5	0	8	0	11	0	29
Coleoptera	Polyphaga	Melyridae	*Dasytes plumbeus*	0	0	0	0	4	0	0	0	4
Coleoptera	Polyphaga	Mordellidae	*Mordellochroa abdominalis*	0	0	1	0	0	0	0	0	1
Coleoptera	Polyphaga	Mordellidae	*Tomoxia bucephala*	0	0	1	0	0	0	0	0	1
Coleoptera	Polyphaga	Mycetophagidae	*Litargus connexus*	3	0	8	0	5	0	3	0	19
Coleoptera	Polyphaga	Mycetophagidae	*Mycetophagus atomarius*	1	0	0	0	1	0	0	0	2
Coleoptera	Polyphaga	Mycetophagidae	*Mycetophagus piceus*	5	0	0	0	0	0	0	0	5
Coleoptera	Polyphaga	Mycetophagidae	*Mycetophagus quadriguttatus*	0	1	0	0	0	0	0	0	1
Coleoptera	Polyphaga	Mycetophagidae	*Mycetophagus quadripustulatus*	4	0	0	0	0	0	0	0	4
Coleoptera	Polyphaga	Nitidulidae	*Cryptarcha undata*	0	0	4	7	0	0	0	0	11
Coleoptera	Polyphaga	Nitidulidae	*Cychramus luteus*	0	0	0	0	2	0	0	0	2
Coleoptera	Polyphaga	Nitidulidae	*Cychramus variegatus*	5	1	2	0	50	6	32	0	96
Coleoptera	Polyphaga	Nitidulidae	*Epuraea marseuli*	0	0	0	0	2	0	0	0	2
Coleoptera	Polyphaga	Nitidulidae	*Epuraea melanocephala*	2	0	0	0	1	0	4	0	7
Coleoptera	Polyphaga	Nitidulidae	*Epuraea pygmaea*	0	0	0	0	27	0	0	0	27
Coleoptera	Polyphaga	Nitidulidae	*Epuraea unicolor*	0	0	0	0	4	0	0	0	4
Coleoptera	Polyphaga	Nitidulidae	*Glischrochilus quadripunctatus*	0	0	0	0	1	0	0	0	1
Coleoptera	Polyphaga	Nitidulidae	*Glischrochilus quadrisignatus*	0	0	0	0	0	0	1	0	1
Coleoptera	Polyphaga	Nitidulidae	*Meligethes aeneus*	1	0	1	0	0	0	0	1	3
Coleoptera	Polyphaga	Nitidulidae	*Meligethes denticulatus*	0	0	0	0	3	0	0	0	3
Coleoptera	Polyphaga	Nitidulidae	*Meligethes nigrescens*	0	0	0	0	1	0	0	0	1
Coleoptera	Polyphaga	Nitidulidae	*Meligethes pedicularius*	0	0	0	0	2	0	0	0	2
Coleoptera	Polyphaga	Nitidulidae	*Pityophagus ferrugineus*	0	0	0	0	2	0	0	0	2
Coleoptera	Polyphaga	Omalisidae	*Omalisus fontisbellaquaei*	0	0	0	0	1	0	0	0	1
Coleoptera	Polyphaga	Pselaphidae	*Bibloporus bicolor*	1	0	0	0	4	0	1	0	6
Coleoptera	Polyphaga	Pselaphidae	*Bibloporus minutus*	0	0	1	0	1	1	0	0	3
Coleoptera	Polyphaga	Pselaphidae	*Brachygluta sinuata*	0	0	0	0	1	0	0	0	1
Coleoptera	Polyphaga	Pselaphidae	*Bryaxis curtisii*	0	0	0	0	1	0	0	0	1
Coleoptera	Polyphaga	Pselaphidae	*Bryaxis nodicornis*	0	0	0	0	1	0	0	0	1
Coleoptera	Polyphaga	Pselaphidae	*Bryaxis puncticollis*	1	0	0	0	0	0	0	0	1

Coleoptera	Polyphaga	Pselaphidae	*Bythinus burrelli*	1	0	1	0	2	0	1	0	5
Coleoptera	Polyphaga	Pselaphidae	*Bythinus macropalpus*	0	0	0	0	1	0	0	0	1
Coleoptera	Polyphaga	Pselaphidae	*Euplectus bescidicus*	5	0	0	0	0	0	0	0	5
Coleoptera	Polyphaga	Pselaphidae	*Euplectus karsteni*	0	0	0	0	0	1	0	0	1
Coleoptera	Polyphaga	Pselaphidae	*Euplectus punctatus*	0	0	0	0	3	0	0	0	3
Coleoptera	Polyphaga	Pselaphidae	*Plectophloeus fischeri*	22	0	2	0	45	1	2	0	72
Coleoptera	Polyphaga	Pselaphidae	*Trichonyx sulcicollis*	1	0	0	0	0	0	0	0	1
Coleoptera	Polyphaga	Pselaphidae	*Trimium brevicorne*	0	0	1	0	0	0	0	0	1
Coleoptera	Polyphaga	Ptiliidae	*Acrotrichis grandicollis*	0	0	1	0	0	0	0	0	1
Coleoptera	Polyphaga	Ptiliidae	*Acrotrichis insularis*	0	0	0	0	1	0	0	0	1
Coleoptera	Polyphaga	Ptiliidae	*Acrotrichis intermedia*	4	0	0	0	7	0	0	0	11
Coleoptera	Polyphaga	Ptiliidae	*Baeocrara variolosa*	3	0	0	0	0	0	0	0	3
Coleoptera	Polyphaga	Ptiliidae	*Ptenidium laevigatum*	0	0	0	0	1	0	0	0	1
Coleoptera	Polyphaga	Ptiliidae	*Pteryx suturalis*	0	0	0	0	0	1	0	0	1
Coleoptera	Polyphaga	Ptinidae	*Ptinus subpilosus*	0	0	0	0	1	0	1	0	2
Coleoptera	Polyphaga	Pyrochroidae	*Schizotus pectinicornis*	0	0	1	0	0	0	0	0	1
Coleoptera	Polyphaga	Rhizophagidae	*Monotoma longicollis*	0	0	0	0	1	0	0	0	1
Coleoptera	Polyphaga	Rhizophagidae	*Rhizophagus bipustulatus*	2	0	0	0	4	0	0	0	6
Coleoptera	Polyphaga	Rhizophagidae	*Rhizophagus depressus*	0	0	0	0	4	0	0	0	4
Coleoptera	Polyphaga	Rhizophagidae	*Rhizophagus dispar*	5	0	0	0	1	0	0	0	6
Coleoptera	Polyphaga	Rhizophagidae	*Rhizophagus ferrugineus*	0	0	0	0	6	0	0	0	6
Coleoptera	Polyphaga	Rhizophagidae	*Rhizophagus nitidulus*	0	0	0	0	1	0	0	0	1
Coleoptera	Polyphaga	Rhizophagidae	*Rhizophagus perforatus*	12	0	0	1	8	0	0	0	21
Coleoptera	Polyphaga	Rhynchitidae	*Caenorhinus germanicus*	0	0	0	0	2	0	0	0	2
Coleoptera	Polyphaga	Salpingidae	*Salpingus planirostris*	9	1	9	0	3	0	0	0	22
Coleoptera	Polyphaga	Salpingidae	*Salpingus ruficollis*	1	0	3	0	6	0	0	0	10
Coleoptera	Polyphaga	Salpingidae	*Vincenzellus ruficollis*	1	0	0	0	0	0	1	0	2
Coleoptera	Polyphaga	Scarabaeidae	*Aphodius constans*	1	0	0	0	2	0	0	0	3
Coleoptera	Polyphaga	Scirtidae	*Cyphon coarctatus*	1	0	0	0	1	0	0	0	2
Coleoptera	Polyphaga	Scolytidae	*Cryphalus piceae*	0	0	1	0	0	0	0	0	1

Coleoptera	Polyphaga	Scolytidae	*Crypturgus cinereus*	2	0	0	0	10	0	1	0	13
Coleoptera	Polyphaga	Scolytidae	*Crypturgus hispidulus*	0	0	0	0	10	0	0	0	10
Coleoptera	Polyphaga	Scolytidae	*Dryocoetes autographus*	1	0	0	0	104	0	3	0	108
Coleoptera	Polyphaga	Scolytidae	*Dryocoetes villosus*	5	1	0	0	1	0	0	0	7
Coleoptera	Polyphaga	Scolytidae	*Ernoporicus fagi*	2	0	28	1	2	0	0	0	33
Coleoptera	Polyphaga	Scolytidae	*Gnathotrichus materiarius*	0	0	0	0	1	0	0	0	1
Coleoptera	Polyphaga	Scolytidae	*Hylastes cunicularius*	1	0	0	0	83	0	1	0	85
Coleoptera	Polyphaga	Scolytidae	*Hylurgops palliatus*	0	0	0	0	6	0	0	0	6
Coleoptera	Polyphaga	Scolytidae	*Ips typographus*	0	0	1	0	15	0	1	0	17
Coleoptera	Polyphaga	Scolytidae	*Leperisinus fraxini*	0	0	3	0	0	0	0	0	3
Coleoptera	Polyphaga	Scolytidae	*Pityogenes chalcographus*	0	0	0	0	25	0	6	0	31
Coleoptera	Polyphaga	Scolytidae	*Pityophthorus exsculptus*	0	0	0	0	0	0	9	0	9
Coleoptera	Polyphaga	Scolytidae	*Pityophthorus pityographus*	0	0	0	0	8	0	17	0	25
Coleoptera	Polyphaga	Scolytidae	*Polygraphus poligraphus*	0	0	0	0	0	0	1	0	1
Coleoptera	Polyphaga	Scolytidae	*Scolytus intricatus*	0	0	0	0	0	0	2	0	2
Coleoptera	Polyphaga	Scolytidae	*Taphrorychus bicolor*	0	0	5	3	1	0	0	0	9
Coleoptera	Polyphaga	Scolytidae	*Xyleborus dispar*	7	0	3	0	30	3	1	0	44
Coleoptera	Polyphaga	Scolytidae	*Xyleborus germanus*	420	3	1	0	226	1	0	0	651
Coleoptera	Polyphaga	Scolytidae	*Xyleborus monographus*	0	0	1	0	0	0	0	0	1
Coleoptera	Polyphaga	Scolytidae	*Xyleborus peregrinus*	0	0	37	2	3	0	3	0	45
Coleoptera	Polyphaga	Scolytidae	*Xyleborus saxeseni*	4	1	28	2	20	0	7	0	62
Coleoptera	Polyphaga	Scolytidae	*Xyloterus domesticus*	3	0	0	0	1	0	0	0	4
Coleoptera	Polyphaga	Scolytidae	*Xyloterus lineatus*	0	0	0	0	4	0	0	0	4
Coleoptera	Polyphaga	Scraptiidae	*Anaspis frontalis*	0	0	0	0	1	1	0	0	2
Coleoptera	Polyphaga	Scraptiidae	*Anaspis ruficollis*	0	0	0	0	1	0	0	0	1
Coleoptera	Polyphaga	Scraptiidae	*Anaspis rufilabris*	0	0	0	0	2	0	5	0	7
Coleoptera	Polyphaga	Scraptiidae	*Anaspis thoracica*	2	0	1	0	1	1	2	0	7
Coleoptera	Polyphaga	Scydmaenidae	*Neuraphes elongatulus*	1	0	1	0	0	0	0	0	2
Coleoptera	Polyphaga	Silphidae	*Oiceoptoma thoracica*	1	0	0	0	0	0	0	0	1
Coleoptera	Polyphaga	Silvanidae	*Silvanoprus fagi*	0	0	0	0	13	0	1	0	14

Coleoptera	Polyphaga	Silvanidae	*Uleiota planata*	0	0	0	0	2	0	0	0	2
Coleoptera	Polyphaga	Staphylinidae	*Aleochara bipustulata*	1	0	1	0	0	0	0	0	2
Coleoptera	Polyphaga	Staphylinidae	*Aleocharinae spec.*	0	0	0	0	1	0	0	0	1
Coleoptera	Polyphaga	Staphylinidae	*Amischa analis*	0	0	0	0	1	0	1	0	2
Coleoptera	Polyphaga	Staphylinidae	*Anotylus tetracarinatus*	1	0	0	0	0	0	0	0	1
Coleoptera	Polyphaga	Staphylinidae	*Atheta aegra*	1	0	0	0	1	0	0	0	2
Coleoptera	Polyphaga	Staphylinidae	*Atheta cauta*	1	0	0	0	0	0	0	0	1
Coleoptera	Polyphaga	Staphylinidae	*Atheta crassicornis*	1	0	0	0	1	0	0	0	2
Coleoptera	Polyphaga	Staphylinidae	*Atheta fungi*	1	0	0	0	5	0	0	0	6
Coleoptera	Polyphaga	Staphylinidae	*Atheta incognita*	0	0	0	0	1	0	0	0	1
Coleoptera	Polyphaga	Staphylinidae	*Atheta myrmecobia*	0	0	0	0	0	1	0	0	1
Coleoptera	Polyphaga	Staphylinidae	*Atheta nigricornis*	2	0	2	0	3	0	0	0	7
Coleoptera	Polyphaga	Staphylinidae	*Atheta palustris*	0	0	0	0	2	0	0	0	2
Coleoptera	Polyphaga	Staphylinidae	*Atheta sodalis*	1	0	0	0	0	0	0	0	1
Coleoptera	Polyphaga	Staphylinidae	*Atheta subtilis*	0	0	0	0	1	0	0	0	1
Coleoptera	Polyphaga	Staphylinidae	*Atrecus affinis*	0	0	0	0	1	0	0	0	1
Coleoptera	Polyphaga	Staphylinidae	*Bolitochara obliqua*	1	0	0	0	0	0	0	0	1
Coleoptera	Polyphaga	Staphylinidae	*Coprophilus striatulus*	1	0	0	0	0	0	0	0	1
Coleoptera	Polyphaga	Staphylinidae	*Enalodroma hepatica*	1	0	0	0	0	0	0	0	1
Coleoptera	Polyphaga	Staphylinidae	*Euryusa castanoptera*	0	0	0	0	1	0	0	0	1
Coleoptera	Polyphaga	Staphylinidae	*Eusphalerum abdominale*	1	0	1	0	0	0	0	0	2
Coleoptera	Polyphaga	Staphylinidae	*Eusphalerum sorbi*	106	53	4	13	135	192	1	1	505
Coleoptera	Polyphaga	Staphylinidae	*Gabrius coxalus*	0	0	0	0	1	0	0	0	1
Coleoptera	Polyphaga	Staphylinidae	*Gabrius splendidulus*	0	0	0	0	1	0	0	0	1
Coleoptera	Polyphaga	Staphylinidae	*Geostiba circellaris*	1	0	0	0	0	0	0	0	1
Coleoptera	Polyphaga	Staphylinidae	*Habrocerus capillaricornis*	0	0	0	0	1	0	0	0	1
Coleoptera	Polyphaga	Staphylinidae	*Haploglossa villosula*	1	0	0	0	3	0	0	0	4
Coleoptera	Polyphaga	Staphylinidae	*Heterothops dissimilis*	0	0	0	0	1	0	0	0	1
Coleoptera	Polyphaga	Staphylinidae	*Holobus flavicornis*	0	0	0	0	0	0	1	0	1
Coleoptera	Polyphaga	Staphylinidae	*Ischnoglossa spec.*	0	0	2	0	0	0	0	0	2

Coleoptera	Polyphaga	Staphylinidae	*Leptusa fumida*	0	0	1	0	0	0	0	0	1
Coleoptera	Polyphaga	Staphylinidae	*Leptusa norvegica*	1	0	0	0	0	0	0	0	1
Coleoptera	Polyphaga	Staphylinidae	*Leptusa pulchella*	1	0	0	0	1	0	0	0	2
Coleoptera	Polyphaga	Staphylinidae	*Leptusa ruficollis*	1	0	0	0	0	0	0	0	1
Coleoptera	Polyphaga	Staphylinidae	*Lesteva longoelytrata*	1	0	0	0	2	0	0	0	3
Coleoptera	Polyphaga	Staphylinidae	*Mycetoporus lepidus*	0	0	0	0	0	0	1	0	1
Coleoptera	Polyphaga	Staphylinidae	*Omalium caesum*	0	0	0	0	1	0	0	0	1
Coleoptera	Polyphaga	Staphylinidae	*Omalium rivulare*	3	0	0	0	0	0	0	0	3
Coleoptera	Polyphaga	Staphylinidae	*Oxypoda alternans*	1	0	0	0	0	0	0	0	1
Coleoptera	Polyphaga	Staphylinidae	*Oxypoda opaca*	0	0	0	0	0	0	1	0	1
Coleoptera	Polyphaga	Staphylinidae	*Philonthus albipes*	1	0	0	0	0	0	0	0	1
Coleoptera	Polyphaga	Staphylinidae	*Philonthus cognatus*	0	0	0	0	1	0	0	0	1
Coleoptera	Polyphaga	Staphylinidae	*Philonthus concinnus*	0	0	1	0	0	0	0	0	1
Coleoptera	Polyphaga	Staphylinidae	*Phloeocharis subtilissima*	1	0	0	0	0	0	0	0	1
Coleoptera	Polyphaga	Staphylinidae	*Phloeopora corticalis*	0	0	5	0	0	0	0	0	5
Coleoptera	Polyphaga	Staphylinidae	*Phloeopora testacea*	0	0	0	0	1	0	0	0	1
Coleoptera	Polyphaga	Staphylinidae	*Phyllodrepa ioptera*	1	0	0	0	0	0	0	0	1
Coleoptera	Polyphaga	Staphylinidae	*Placusa tachyporoides*	0	0	0	0	3	0	0	0	3
Coleoptera	Polyphaga	Staphylinidae	*Quedius maurus*	1	0	0	0	0	0	0	0	1
Coleoptera	Polyphaga	Staphylinidae	*Quedius mesomelinus*	1	0	0	0	0	0	0	0	1
Coleoptera	Polyphaga	Staphylinidae	*Quedius xanthopus*	0	1	0	0	5	0	0	0	6
Coleoptera	Polyphaga	Staphylinidae	*Rugilus rufipes*	0	0	0	0	0	1	0	0	1
Coleoptera	Polyphaga	Staphylinidae	*Scaphisoma agaricinum*	3	0	0	0	0	0	0	0	3
Coleoptera	Polyphaga	Staphylinidae	*Scopaeus laevigatus*	0	0	0	0	0	0	1	0	1
Coleoptera	Polyphaga	Staphylinidae	*Syntomium aeneum*	1	0	0	0	0	0	0	0	1
Coleoptera	Polyphaga	Staphylinidae	*Tachinus elongatus*	1	0	0	0	0	0	0	0	1
Coleoptera	Polyphaga	Staphylinidae	*Tachinus signatus*	1	0	0	0	0	0	0	0	1
Coleoptera	Polyphaga	Tenebrionidae	*Bolitophagus reticulatus*	2	0	0	0	1	0	0	0	3
Coleoptera	Polyphaga	Tenebrionidae	*Stenomax aeneus*	0	0	1	0	3	0	0	0	4
Coleoptera	Polyphaga	Throscidae	*Aulonothroscus brevicollis*	1	0	0	0	3	1	0	1	6

Coleoptera	Polyphaga	Throscidae	*Trixagus dermestoides*	2	2	1	0	96	43	6	2	152
Coleoptera	Polyphaga	Throscidae	*Trixagus leseigneuri*	0	0	0	0	7	2	0	0	9
Coleoptera	Polyphaga	Throscidae	*Trixagus meybohmi*	0	0	0	0	1	4	0	0	5
Coleoptera	Polyphaga	Trogidae	*Trox scaber*	0	0	1	0	0	0	0	0	1
Coleoptera	Polyphaga	Trogositidae	*Nemosoma elongatum*	0	0	2	1	5	0	1	0	9
Hemiptera	Heteroptera	Acanthosomatidae	*Acanthosoma haemorrhoidale*	0	0	1	0	0	0	0	0	1
Hemiptera	Heteroptera	Anthocoridae	*Anthocoris confusus*	1	0	1	1	0	0	0	0	3
Hemiptera	Heteroptera	Anthocoridae	*Orius majusculus*	0	0	1	0	0	0	0	0	1
Hemiptera	Heteroptera	Anthocoridae	*Orius minutus*	0	0	1	0	0	1	0	0	2
Hemiptera	Heteroptera	Anthocoridae	*Temnostethus pusillus*	0	0	1	0	0	0	0	0	1
Hemiptera	Heteroptera	Aradidae	*Aradus depressus*	1	0	0	0	0	0	0	0	1
Hemiptera	Heteroptera	Lygaeidae	*Drymus ryeii*	0	0	0	0	1	0	0	0	1
Hemiptera	Heteroptera	Lygaeidae	*Gastrodes abietum*	0	0	0	0	2	1	2	0	5
Hemiptera	Heteroptera	Lygaeidae	*Kleidocerys resedae*	0	0	1	0	1	0	0	0	2
Hemiptera	Heteroptera	Lygaeidae	*Trapezonotus dispar*	0	0	0	0	1	0	0	0	1
Hemiptera	Heteroptera	Microphysidae	*Loricula elegantula*	0	0	2	0	2	0	1	1	6
Hemiptera	Heteroptera	Microphysidae	*Loricula pselaphiformis*	5	0	1	0	0	0	0	0	6
Hemiptera	Heteroptera	Miridae	*Actinonotus pulcher*	0	0	0	0	2	0	0	0	2
Hemiptera	Heteroptera	Miridae	*Atractotomus magnicornis*	0	0	0	0	5	0	12	0	17
Hemiptera	Heteroptera	Miridae	*Blepharidopterus angulatus*	1	1	0	0	0	0	0	0	2
Hemiptera	Heteroptera	Miridae	*Campyloneura virgula*	0	2	0	0	0	0	0	0	2
Hemiptera	Heteroptera	Miridae	*Cremnocephalus alpestris*	0	0	0	0	0	0	7	0	7
Hemiptera	Heteroptera	Miridae	*Deraeocoris lutescens*	0	0	1	0	0	0	0	0	1
Hemiptera	Heteroptera	Miridae	*Dryophilocoris flavoquadrimaculatus*	6	1	1	0	0	0	0	0	8
Hemiptera	Heteroptera	Miridae	*Harpocera thoracica*	0	0	0	0	1	0	4	0	5
Hemiptera	Heteroptera	Miridae	*Monalocoris filicis*	0	0	0	0	0	1	0	0	1
Hemiptera	Heteroptera	Miridae	*Parapsallus vitellinus*	0	0	0	0	4	0	9	2	15
Hemiptera	Heteroptera	Miridae	*Phylus melanocephalus*	1	0	0	0	0	0	0	0	1
Hemiptera	Heteroptera	Miridae	*Phytocoris dimidiatus*	0	0	4	0	0	0	0	0	4
Hemiptera	Heteroptera	Miridae	*Phytocoris longipennis*	0	0	0	0	0	4	0	1	5

Hemiptera	Heteroptera	Miridae	*Psallus varians*	10	1	23	0	1	0	0	0	35
Hemiptera	Heteroptera	Miridae	*Rhabdomiris striatellus*	0	1	2	0	0	0	0	0	3
Hemiptera	Heteroptera	Nabidae	*Himacerus apterus*	2	0	1	0	1	0	1	0	5
Hemiptera	Heteroptera	Pentatomidae	*Chlorochroa pinicola*	0	0	0	0	0	0	1	0	1
Hemiptera	Heteroptera	Pentatomidae	*Dolycoris baccarum*	0	0	1	0	0	0	0	0	1
Hemiptera	Heteroptera	Pentatomidae	*Palomena prasina*	0	0	0	0	1	0	0	0	1
Hemiptera	Heteroptera	Pentatomidae	*Pentatoma rufipes*	5	0	6	1	3	0	3	0	18
**Total**				**1241**	**116**	**629**	**88**	**2202**	**347**	**777**	**42**	**5442**
