# Supplementary material for: Searching for the Optimal Sampling Solution: Variation in Invertebrate Communities, Sample Condition and DNA Quality
Source: PLoS One. 2016 Feb 3;11(2):e0148247. doi: 10.1371/journal.pone.0148247 (PMC4740435; doi:10.1371/journal.pone.0148247)
Supplement: S8 File — Fig A shows the probability of successful ID of a sample as a function of the concentration of good DNA in the sample. Fig B shows the probability of successful ID of a sample as a function of the % of good DNA in the sample. (PDF) [file pone.0148247.s008.pdf]

Supplementary S8: Additional information on DNA barcoding

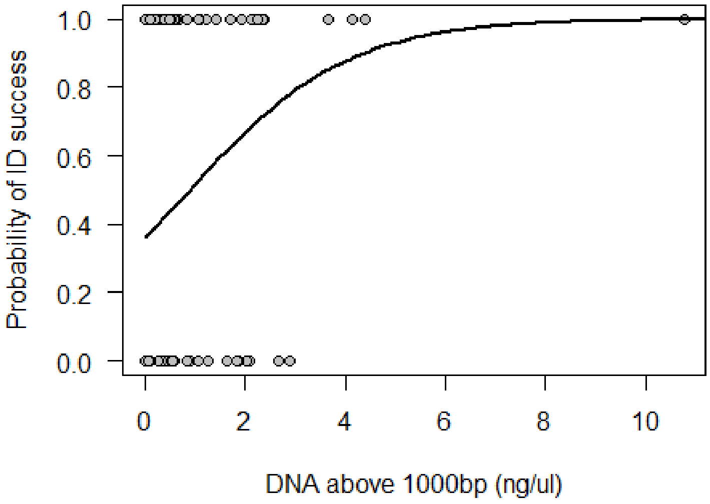

Figure A: The probability of successful ID of a sample as a function of the concentration of good DNA in the sample.

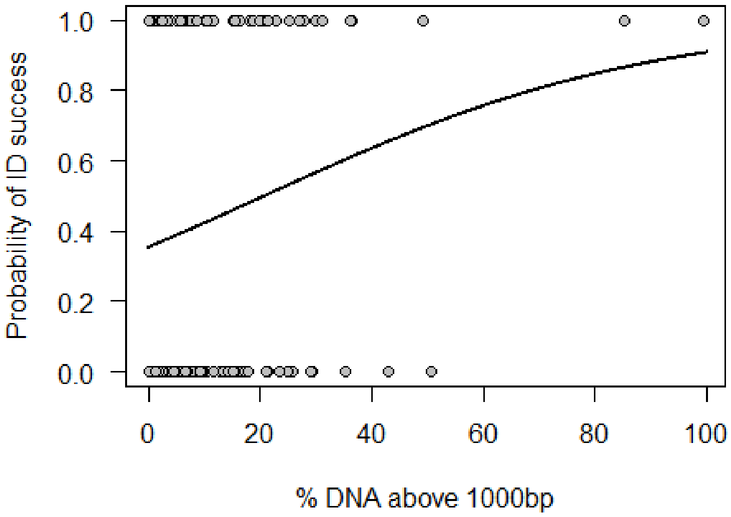

Figure B: The probability of successful ID of a sample as a function of the % of good DNA in the sample.
